# Generating intravital super-resolution movies with conventional microscopy reveals actin dynamics that construct pioneer axons

**DOI:** 10.1242/dev.171512

**Published:** 2019-03-08

**Authors:** Yide Zhang, Evan L. Nichols, Abigail M. Zellmer, Ian H. Guldner, Cody Kankel, Siyuan Zhang, Scott S. Howard, Cody J. Smith

**Affiliations:** 1 Department of Electrical Engineering, University of Notre Dame, Notre Dame, IN 46556, USA; 2 Department of Biological Sciences, University of Notre Dame, Notre Dame, IN 46556, USA; 3 Center for Stem Cells and Regenerative Medicine, University of Notre Dame, Notre Dame, IN 46556, USA; 4 Mike and Josie Harper Cancer Research Institute, University of Notre Dame, Notre Dame, IN 46556, USA; 5 Indiana University Melvin and Bren Simon Cancer Center, Indianapolis, IN 46202, USA; 6 Center for Research Computing. University of Notre Dame, Notre Dame, IN 46556, USA

**Keywords:** Super-resolution, Neurodevelopment, DeSOS

## Abstract

Super-resolution microscopy is broadening our in-depth understanding of cellular structure. However, super-resolution approaches are limited, for numerous reasons, from utilization in longer-term intravital imaging. We devised a combinatorial imaging technique that combines deconvolution with stepwise optical saturation microscopy (DeSOS) to circumvent this issue and image cells in their native physiological environment. Other than a traditional confocal or two-photon microscope, this approach requires no additional hardware. Here, we provide an open-access application to obtain DeSOS images from conventional microscope images obtained at low excitation powers. We show that DeSOS can be used in time-lapse imaging to generate super-resolution movies in zebrafish. DeSOS was also validated in live mice. These movies uncover that actin structures dynamically remodel to produce a single pioneer axon in a ‘top-down’ scaffolding event. Further, we identify an F-actin population – stable base clusters – that orchestrate that scaffolding event. We then identify that activation of Rac1 in pioneer axons destabilizes stable base clusters and disrupts pioneer axon formation. The ease of acquisition and processing with this approach provides a universal technique for biologists to answer questions in living animals.

## Abstract (precis)

**Summary:** Actin dynamics are examined in zebrafish axons using DeSOS, a new super-resolution technique combining deconvolution with stepwise optical saturation microscopy that allows detailed intravital imaging of cells in their native environments.

## INTRODUCTION

Cells in living organisms exist in complex and packed environments with unique extracellular milieu next to cells of diverse morphologies and molecular profiles. Biological investigations into the native physiological state of cells often rely on microscopy to visualize cells within organisms. However, conventional (confocal and two-photon) microscopy is limited in its ability to resolve some subcellular structures owing to the optical diffraction limit (Cole et al., 2011).

To overcome the diffraction limit, super-resolution microscopy has been developed to reveal the detailed organization of subcellular structures. Optical super-resolution [e.g. stimulated emission depletion (STED), photoactivated localization (PALM), stochastic optical reconstruction (STORM) and structured illumination (SIM) microscopy] uses enhanced imaging systems to acquire images of biological specimens (Betzig et al., 2006; Gustafsson, 2005; Hell and Wichmann, 1994; Rust et al., 2006; Strauss et al., 2012). Most optical super-resolution microscopes require thin slices of tissue, so cells must be removed from their native environment for imaging. Although STED permits 3D imaging in tissues, its penetration depth is limited because scattering and aberrations in tissues can break the alignment of the two lasers, degrading image quality (Vicidomini et al., 2018). Alternatively, optical techniques generally use extensive laser powers or exposure times within tissue, limiting longer-term imaging. Super-resolution radial fluctuations (SRRF) reduces laser power and exposure but requires extensive computing power and is not compatible for use in three-dimensional samples. Recently, lattice light sheet microscopy was combined with optical imaging to provide super-resolution intravital protein organization over extended periods of time (Liu et al., 2018). Collectively, such optical techniques require the acquisition of expensive hardware. Expansion microscopy cross-links antibodies or fluorophores to a polymer gel, which then is expanded, to provide super-resolution (Chen et al., 2015; Chozinski et al., 2016; Freifeld et al., 2017). The advantage to expansion microscopy is its performance in tissue environments and whole animals with the use of conventional microscopes. However, expansion microscopy requires fixation and thus cannot be used in living animals (Chozinski et al., 2016; Freifeld et al., 2017). Together, these approaches, unfortunately, are limited in their ability to image cells in their native environment over extended periods of time in volume stacks.

Here, we begin to answer these questions by developing an intravital super-resolution technique, DeSOS, combining stepwise optical saturation super-resolution (SOS) and blind deconvolution (Holmes et al., 2006; Zhang et al., 2018). With DeSOS, we can obtain intravital super-resolution movies using conventional microscopy. Here, we provide an open-access application to obtain DeSOS images. We validate DeSOS by imaging actin arrangement in dorsal root ganglia (DRG) neurons in zebrafish via time-lapse movies, identifying seven distinct actin subpopulations in developing neurons. Our data show that two of these subpopulations orchestrate the production and selection of the pioneer axon. We then identify Rac1 as a key regulator of the dynamics of these subpopulations. Overall, we introduce DeSOS as an accessible technique to acquire super-resolution images of cells in their native environments.

## RESULTS

### Principle of DeSOS

**Fig. 1.**
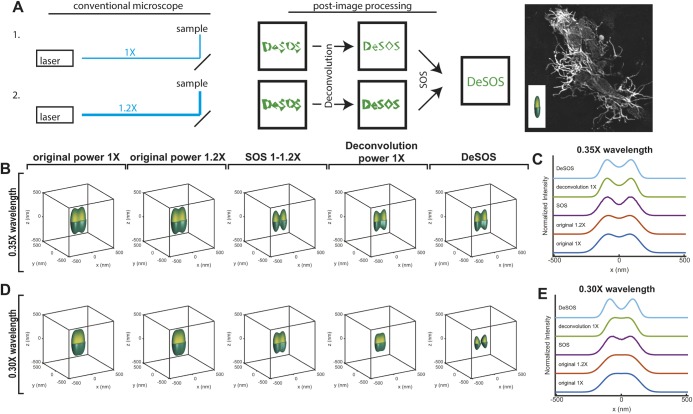
**Principle of DeSOS.** (A) In DeSOS, two conventional (confocal or two-photon) fluorescence images obtained with different laser powers (e.g. 1× and 1.2× the original power) are processed with blind deconvolution and SOS, to generate a DeSOS image. As an example, the *z*-projection of a DeSOS image of a *Tg(sox10:gal4);* *Tg(uas:lifeact-GFP)* transgenic zebrafish fixed at 54 hpf, with its PSF, is shown. (B) Simulated conventional (1× and 1.2× laser powers), SOS, blind deconvolution, and DeSOS images of two adjacent point objects where their distance is 0.35× the excitation wavelength. (C) Line profiles of the simulated images in B in the lateral direction (*y*=0, *z*=0). (D) Simulated images of the two point objects as in B separated by 0.30× the excitation wavelength. (E) Line profiles of the simulated images in D in the lateral direction.

To acquire super-resolution time-lapse movies in tissue samples at least 40 µm deep, we sought a super-resolution technique that used low laser powers and short acquisition times. SOS, a saturation-based super-resolution fluorescence microscopy technique, allows users to obtain super-resolution images with conventional confocal or two-photon microscopes. Using linear combination of two conventional fluorescence images obtained with different illumination powers (e.g. 1× and 1.2× the original power), SOS increases image resolution 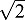-fold. The ratio of the two laser powers does not need to be exactly 1.2; it can be any number larger than 1, but ∼1.2 is recommended for optimal signal-to-noise ratio (SNR) performance (Zhang et al., 2018). A two-step SOS, however, could reduce the SNR of the raw images by an order of magnitude. To alleviate the SNR problem, we hypothesized that deconvolution algorithms could increase the SNR of raw images before SOS. We implemented this approach using blind deconvolution, which utilized an iterative process to produce deconvolved images through a reconstructed point spread function (PSF) estimate capable of adapting to the heterogeneous environment in biological samples without prior knowledge of the PSF (Holmes et al., 2006). Compared with conventional deconvolution methods, which utilize theoretical or measured PSFs of the imaging system, blind deconvolution is superior because it adapts to the real PSF of the microscope and sample, which can be substantially different from theoretical or measured PSFs owing to instrument and sample variations. Unless otherwise noted, all deconvolution terms in this paper refer to blind deconvolution. In confocal and two-photon microscopy, Poisson noise is the major noise source that not only obscures the real structures in the image but also creates impossible features that are smaller than the PSF. Deconvolution amplifies features corresponding to the real structure while rejecting noise features smaller than the PSF, hence increasing the SNR of the raw images. Linear combination of the deconvolved images with the coefficients determined by the excitation laser powers used to obtain the raw images provides the theoretical basis of deconvolution with SOS (DeSOS). For DeSOS microscopy performed on a confocal microscope, for example, if the raw images *F*_1_ and *F*_2_ are obtained with a laser power of *I*_1_=1 mW and *I*_2_=1.2 mW, respectively, the linear combination coefficients will be calculated as *c*_1_=1, *c*_2_=−*I*_1_/*I*_2_=−0.83 according to the SOS algorithm (Zhang et al., 2018), and the resulting super-resolution DeSOS image will be *c*_1_Decon[*F*_1_]+*c*_2_Decon[*F*_2_]=Decon[*F*_1_]−0.83Decon[*F*_2_] (Fig. 1A).

**Fig. 2.**
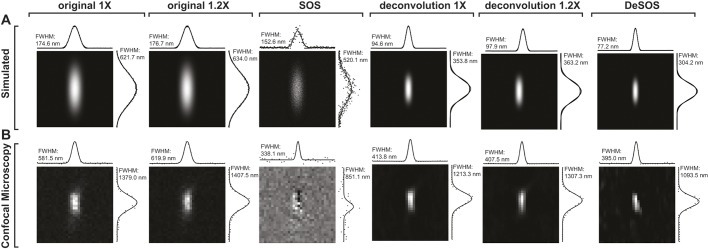
**Simulated PSFs and images of a fluorescent bead.** (A) Theoretically simulated PSFs of conventional, SOS, deconvolution and DeSOS images. Line profiles of the PSFs in lateral and axial directions have been plotted and fitted to a Gaussian function. FWHM is shown. (B) Confocal, SOS, deconvolution and DeSOS images of a 500 nm fluorescent bead. Line profiles of the bead images in lateral and axial directions have been plotted and fitted to a Gaussian function. FWHM is presented.

The most important advantage of DeSOS is that its resolution is higher than conventional and deconvolved images and its SNR performance is substantially better than SOS. As can be seen from theoretically simulated PSFs of conventional, SOS, deconvolved and DeSOS images (Fig. 2A), although SOS and DeSOS both show a reduction in the PSF's full width at half maximum (FWHM) compared with conventional PSFs, SOS's SNR is substantially lower than that of DeSOS. The deconvolved PSFs also show an increase in SNR and apparent reduction in the PSF's FWHMs. Among all PSFs, the best performance with respect to both resolution and SNR is provided by the DeSOS PSF with a lateral FWHM of 77.2 nm and an axial FWHM of 304.2 nm, a 2.26-fold and 2.04-fold increase in lateral and axial resolutions, respectively, compared with the conventional PSF. Note that although the reduction in FWHMs can also be seen in the deconvolved PSFs, this was mainly due to its higher contrast by reducing optical distortion and did not indicate that deconvolution physically provided better resolution (MacDonald et al., 2015; McNally et al., 1999). To demonstrate this further, we performed a simulation where two point objects were located next to each other (Fig. 1B,D). As the distance between the centers of these two objects varies, the line profiles in the lateral direction show very distinct features (Fig. 1C,E). When the distance is 0.35× or greater than the excitation wavelength, the two adjacent points are differentiable in the profiles of all image modalities, including the original and deconvolution ones; moreover, deconvolution demonstrates similar differentiability compared with SOS and DeSOS. However, when the distance is closer, e.g. 0.30× the wavelength, the simulations demonstrate that the adjacent points become differentiable only in SOS and DeSOS modalities.

To test these simulations, we compared the theoretical PSFs in parallel to fluorescent images obtained with confocal microscopy (Fig. 2). For this comparison, we imaged a 500 nm fluorescent bead, which was large enough to be properly sampled by our spinning disk confocal microscope with 206 nm minimum pixel size yet small enough to observe resolution performance from lateral and axial FWHMs (Fig. 2B). Compared with the conventional image, the SOS image showed a reduction in bead size with significant SNR degradation. Deconvolved images improved both the SNR and contrast of the image. The best imaging performance was again seen in DeSOS. For a 500 nm object that was conventionally imaged with a lateral FWHM of 581.5 nm and an axial FWHM of 1379.0 nm, the DeSOS image provided a closer representation of its actual size, with lateral and axial FWHMs of 395.0 nm and 1093.5 nm, respectively. Compared with the SOS image, the improvement in SNR performance of the DeSOS image was consistent with theoretical simulations.

### DeSOS produces super-resolution images in intact animals

**Fig. 3.**
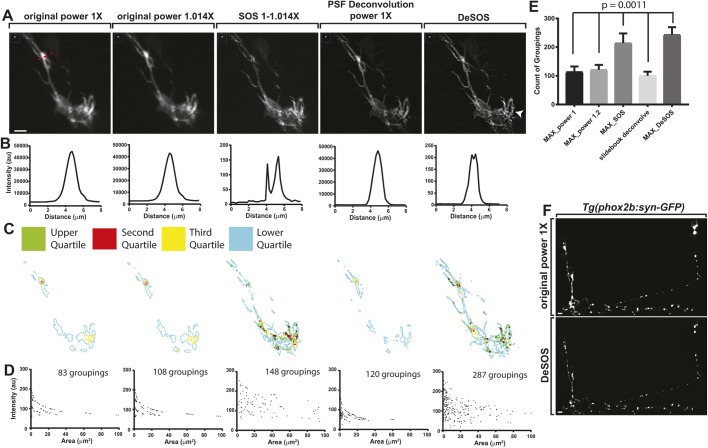
**DeSOS produces super-resolution images in intact animals.** (A) Confocal *z*-projection images of a *Tg(sox10:gal4);* *Tg(uas:lifeact-GFP)* transgenic zebrafish fixed at 54 hpf, visualizing actin within a DRG neuron showing improvement with DeSOS. Dashed red line denotes the ROI used. Images were first taken at laser powers 1× (1.011 mW) and 1.014× (1.025 mW) and processed for SOS, blind deconvolution, and DeSOS. Arrowhead indicates actin cytoskeleton in the cell body. (B) Graphs of pixel intensity across the dashed red line in A showing improved resolution with DeSOS. (C) Pixels in each image subdivided into quartiles by intensity. Composite outlines of each quartile are shown. (D) Adjacent pixels with intensity values within a 10% range of the maximum pixel intensity were grouped and counted for each image and represented by area and mean intensity showing improved resolution in DeSOS. (E) Number of distinct groupings of adjacent pixels from each image (*n*=5 DRG) demonstrating improved resolution of DeSOS (paired one-way ANOVA). (F) *z*-projection confocal image taken at 1.011 mW laser power and its corresponding DeSOS image of a *Tg(phox2b:gal4); Tg(uas:syn-GFP)* zebrafish fixed at 54 hpf showing improved resolution of synapses in the zebrafish brain in the DeSOS image. Scale bars: 10 μm.

We next hypothesized that DeSOS could be used to generate intravital super-resolution images as it only requires standard confocal microscope images with small differences in excitation powers. To test this, we first imaged actin arrangement in DRG neurons in zebrafish fixed at 2 days post-fertilization (dpf) using *Tg(sox10:gal4); Tg(uas:lifeact-GFP)* animals, which use the *sox10* promoter to express Gal4 to drive Lifeact-GFP expression in DRG neurons (Fig. 3A) (Helker et al., 2013; Hines et al., 2015). To demonstrate the utility of DeSOS, we imaged *z*-stacks of the DRG neurons using incremental laser powers: 1.011 mW (1×) and 1.025 mW (1.014×). Stacks were then processed for SOS, deconvolution and DeSOS. SOS images show a noticeable increase in spatial resolution but a low SNR, whereas deconvolution improved the SNR (Fig. 3A). As expected, DeSOS images combined the advantages of SOS and deconvolution individually. To quantify resolution, a region of interest (ROI) with concentrated Lifeact-GFP was analyzed by measuring pixel intensity across the dashed red line in Fig. 3A (Fig. 3B). In the raw confocal images, the width of the peak was 4.5 µm. This decreased significantly in SOS, deconvolution and DeSOS images to 3.0 µm. Further, both SOS and DeSOS distinguished two distinct Lifeact-GFP peaks within the ROI whereas deconvolution alone was unable to do so. To examine the enhanced DeSOS image in more detail, we used ImageJ to subdivide every pixel of the image into quartiles by intensity. We then rendered outlines of each quartile and overlaid them to visualize distinct populations based on intensity. Composite images show that the DeSOS image has greater range and diversity of actin populations (Fig. 3C).

Super-resolution is distinguishable from deconvolution because it can discriminate a single point from two adjacent points. To investigate whether DeSOS could provide super-resolution information across multiple ROIs, we quantified groups of adjacent pixels based on similar intensity values. This analysis used ImageJ to group adjacent pixels that had intensity values within a range of values calculated as 10% of the maximum intensity value in the image. The area and average intensity were measured in each pixel grouping. To calibrate this measurement of super-resolution information, we applied it to the simulated images of adjacent objects separated by 0.3× of the wavelength (Fig. 1D). These measurements demonstrate that SOS and DeSOS provided more pixel groups, consistent with their ability to provide super-resolution information, and, importantly, DeSOS identified the greatest number of groupings (Fig. S1). We next performed these quantifications on multiple ROIs across multiple cell types from our fixed tissue images. In the raw confocal images, distinct groups could be identified at a low frequency, 113.4±19.3 and 121.2±16.9 groupings, respectively (Fig. 3D,E; *n*=5 DRG). SOS demonstrated an increase in the number of individual groups: 213.8±33.7 (Fig. 3D,E; *n*=5 DRG). Deconvolution, consistent with its theoretical inability to increase resolution, had similar pixel groups as the original confocal images (100.2±14.2 groupings) (Fig. 3D,E; *n*=5 DRG). DeSOS demonstrated not only the most groupings of similar pixels with 243.2±26.1 but also greater SNR (Fig. 3D,E; *n*=5 DRG; paired one-way ANOVA, *P*=0.0011). Based on these results, we propose that DeSOS provides super-resolution images in intact animals from images from traditional microscopes.

We next attempted to perform two-color DeSOS with *Tg(sox10:mrfp); Tg(sox10:lifeact-gfp)* animals (Kucenas et al., 2008). In these images, we could visualize membrane labeled with RFP and actin labeled with GFP inside that membrane (Fig. S2A). We further visualized detailed retinal organization with *Tg(sox10:mrfp)* (Fig. S2B). We also used *Tg(phox2b:gal4); Tg(uas:syn-gfp)* to determine whether synaptic vesicles could be visualized at higher resolution with DeSOS (Heap et al., 2013; Hines et al., 2015). Consistent with DeSOS’ widespread utility, synaptic vesicles were clearly labeled, with adjacent synaptic vesicles clearly distinguishable (Fig. 3F). These data are consistent with the hypothesis that DeSOS provides enhanced imaging information for analysis in deep tissue samples.

### Super-resolution DeSOS in living tissue

**Fig. 4.**
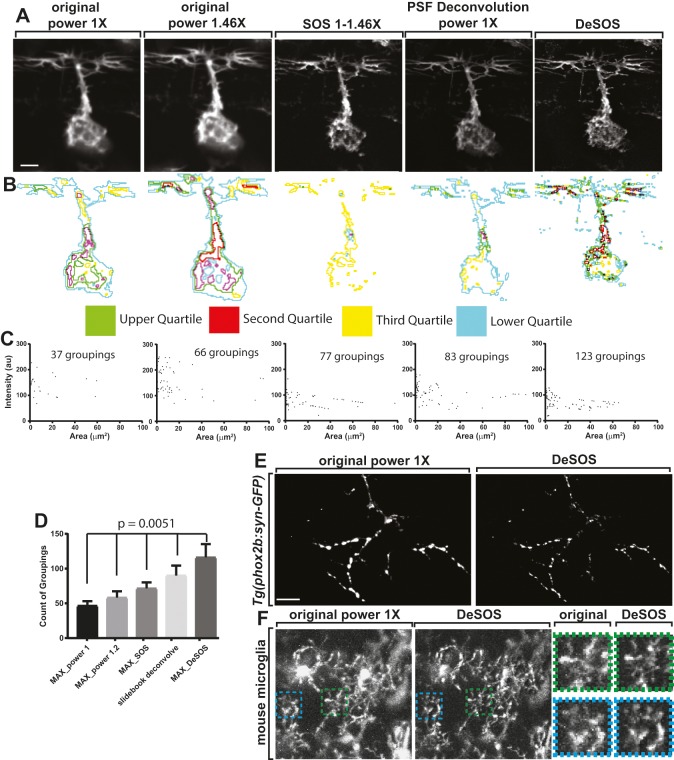
**DeSOS produces super-resolution images in live animals.** (A) Confocal *z*-projection images at 48 hpf of living *Tg(sox10:gal4);* *Tg(uas:lifeact-GFP)* animals. Images were taken at power 1× (59.1 μW), and at laser power 1.46× (86.3 μW). *z*-stacks were processed using SOS, deconvolution and DeSOS. (B) Pixels in each image subdivided into quartiles by intensity to visualize distinct populations of pixels. Composite outlines of each quartile are shown. (C) Adjacent pixels with intensity values within a 10% range of the maximum pixel intensity were grouped. The number of groupings for each image, represented by area and mean intensity are shown. DeSOS discerns additional groups compared with other modalities. (D) Number of distinct pixel groupings from each image (*n*=5 DRG) (paired one-way ANOVA). (E) Confocal *z*-projection images at 48 hpf of living *Tg(phox2b:gal4); Tg(uas:syn-GFP)* animals processed using DeSOS to visualize synaptic vesicles. (F) *z*-projections of a live mouse brain showing microglia with two-photon microscopy and DeSOS. Insets in corresponding colors demonstrate resolution improvement for visualization of microglia processes. Scale bars: 10 μm.

As DeSOS could be applied to fixed zebrafish, we sought to test it in live *Tg(sox10:gal4); Tg(uas:lifeact-GFP)* larvae at 48 hours post-fertilization (hpf) with imaging at sequential laser powers. Because living tissue is sensitive to high laser powers, we utilized lower laser powers in live animals (59.1 µW and 86.3 µW) and repeated the above experiments to determine the potential use of DeSOS in live tissue (Fig. 4A,B). First, by grouping pixels with similar intensities in distinct ROIs, we again measured a difference in the number of unique groupings between each type of image. The unprocessed images showed 46.6±6.5 and 58.2±9.1 distinct groupings (Fig. 4C,D; *n*=5 DRG). SOS increased the number of groupings to 71.4±8.8, particularly visualizing groups with smaller areas (Fig. 4C,D; *n*=5 DRG). DeSOS addressed both issues by increasing the number of distinct pixel groupings (116.0±19.2 groupings; *n*=5 DRG; paired one-way ANOVA, *P*=0.0051) and the SNR (Fig. 4C,D). As with fixed tissue, DeSOS could be used to increase resolution of multicolor zebrafish images as well as distinct protein-labeled synaptic vesicles (Fig. 4E, Fig. S2C,D). Lastly, to demonstrate the universality of DeSOS, we performed it on live mouse brains via cranial windows and two-photon microscopy. In this analysis, microglia processes could be visualized at higher resolution than that achieved by standard two-photon microscopy (Fig. 4F). These results are consistent with the hypothesis that DeSOS allows greater subcellular resolution of structures in living animals.

### Using distinct deconvolution methods to improve signal-to-noise ratio

Many programs for image deconvolution, like AutoQuant Blind, are available for commercial purchase. However, because blind deconvolution algorithms use estimations of the PSF to reverse optical distortion, we predicted that new or publicly available deconvolution algorithms could satisfactorily increase the SNRs of the raw images. To test this, we first used the ImageJ plugin DeconvolutionLab2 (Sage et al., 2017) to deconvolve a *z*-stack of raw images of DRG neurons in Fig. 3. We also developed an open-access DeSOS program that includes a blind deconvolution algorithm implemented with Matlab (MathWorks) to process the raw images (see supplementary Materials and Methods for links to downloadable executable files). We then quantified the groupings of pixels with similar intensities as in Fig. 3. Images processed by the freely available DeconvolutionLab2 and our open-access DeSOS deconvolution algorithms showed enhancement in SNR comparable to that obtained using the AutoQuant Blind program (Fig. S3).

### Comparison of DeSOS and SRRF images of living animals

We also wanted to compare the resolution improvement of DeSOS and another super-resolution technique that requires a conventional microscope, SRRF. To do this, we first took images of the nascent DRG in *Tg(sox10:gal4); Tg(uas:lifeact-gfp)* animals at 1× and 1.46× laser powers for DeSOS processing. We then used the 1× laser to take 100 frames of the same structures to generate a SRRF image (Fig. S4). As in our previous images, DeSOS was able to visualize new information by resolving single points in the raw image into multiple points. In comparison, SRRF also accomplished this and identified bright puncta, consistent with its vector-based identification of local fluorescent maxima. When DeSOS and SRRF images were overlaid, we observed that DeSOS retained a web-like signal in the DRG soma whereas that signal was lost in SRRF. We conclude that the qualitative image provided by SRRF is greatly suited for use in protein puncta localization, and that DeSOS generates a clearer image of complex structures, such as the actin cytoskeleton.

### An open-access DeSOS application

We sought to generate an application that makes DeSOS highly accessible with a standalone program with graphical user interfaces (GUI). The program was implemented with Matlab and can be installed on a Windows PC. A detailed tutorial is presented in supplementary Materials and Methods. To perform DeSOS, the user is asked to input the optical parameters used for acquiring the raw images in order to perform blind deconvolution efficiently. The program uses the input optical parameters to calculate a theoretical PSF to generate an initial estimate as accurately as possible. Next, blind deconvolution is performed on the raw images. After deconvolution, the user can perform the DeSOS algorithm using the GUI.

### Intravital DeSOS movies of developing animals

**Fig. 5.**
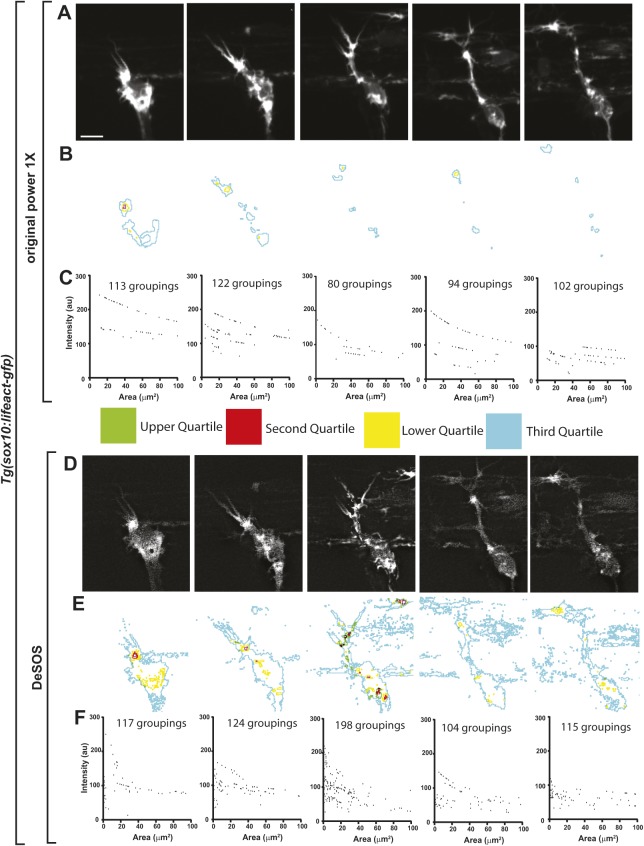
**DeSOS produces super-resolution time-lapse movies.** (A) Confocal *z*-projection images of a *Tg(sox10:lifeact-GFP)* animal from a time-lapse movie with *z*-stacks every 5 min for 24 h starting at 48 hpf. (B) Pixels subdivided into quartiles by intensity to visualize distinct populations of pixels. Composite outlines of each quartile are shown. (C) Adjacent pixels with intensity values within a 10% range of maximum pixel intensity grouped. Number of groupings for each image are represented by area and mean intensity. (D) Confocal *z*-projection images of a *Tg(sox10:lifeact-GFP)* animal from the time-lapse movie shown in A after DeSOS processing. (E) Pixels in each image were subdivided into quartiles as in B. Composite outlines of each quartile are shown. (F) Adjacent pixels for each image in D were grouped as in C and represented by area and mean intensity. Scale bar: 10 μm.

We next investigated whether DeSOS could be used to generate intravital super-resolution time-lapse movies, again using *Tg(sox10:gal4); Tg(uas:lifeact-GFP)* animals. *z*-stacks were taken every 5 min for 24 h at 59.1 µW and 86.3 µW. Laser powers were kept low to avoid phototoxicity and photobleaching. To examine the difference between standard confocal images and DeSOS, we compared individual time points (Fig. 5, Movies 1, 2). The pixel grouping method indicates a consistent increase in the number of identified groupings (Fig. 5C,F). Because DeSOS only requires a small increase in laser power, the changes in overall intensity were minimal. We also did not observe any abnormal death of the animal or cells, both of which would have been signs of phototoxicity. Together, these results support the conclusion that DeSOS can generate intravital super-resolution movies in living tissue samples.

### Super-resolution movies reveal distinct actin dynamics

The viability of DeSOS to create super-resolution intravital movies motivated us to uncover new biology by analyzing our DeSOS movies. During this time in development (48-72 hpf), DRG pioneer axons initiate dorsally to the dorsal root entry zone (DREZ), enter the spinal cord, and then form branches that extend anteriorly and posteriorly (Nichols and Smith, 2019; Smith et al., 2017). Although these processes have been identified in other experimental paradigms, super-resolution imaging of dynamic actin arrangements during the totality of these events has not been pursued in living animals. We sought to identify distinct actin populations in intravital DeSOS movies using four parameters: temporal stability, pixel intensity, size and shape, and location in the neuron.

**Fig. 6.**
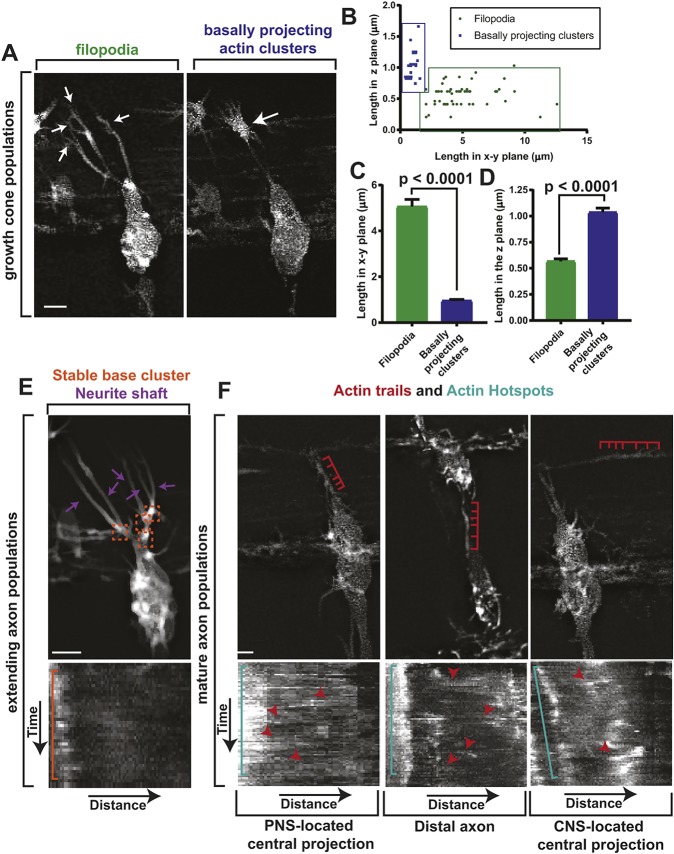
**DeSOS visualizes at least seven distinct actin populations.** (A) Confocal *z*-projection DeSOS images of a *Tg(sox10:gal4);* *Tg(uas:lifeact-GFP)* zebrafish showing growth cone actin dynamics. White arrows denote populations of interest. (B) Length of filopodia (green) and basally projecting clusters (blue) in the *xy*-plane and *z*-plane. *n*=44 filopodia, *n*=16 basally projecting clusters. (C) Length of filopodia and basally projecting clusters in the *xy*-plane. *n*=44 filopodia, *n*=16 basally projecting clusters. Student's *t*-test. (D) Length of filopodia projections and basally projecting clusters in the *z*-plane. *n*=44 filopodia, *n*=16 basally projecting clusters. Student's *t*-test. (E) Confocal *z*-projection DeSOS image of a *Tg(sox10:gal4);* *Tg(uas:lifeact-GFP)* zebrafish showing actin dynamics in the extending axon. Purple arrows denote neurite shafts. Orange boxes denote SBCs. Kymograph shows SBC stability (orange bracket). (F) Confocal *z*-projection DeSOS images of a *Tg(sox10:gal4);* *Tg(uas:lifeact-GFP)* zebrafish showing actin dynamics in mature axons. Red brackets denote actin trails. Kymograph shows actin hotspot (teal brackets) and actin trails (red arrowheads). Scale bars: 10 μm.

We first observed actin dynamics at the growth cone. One population consisted of thin actin protrusions that extended from the central growth cone and were present as the axon navigated to the DREZ and after the axon entered the spinal cord (Fig. 6A). However, they were absent for ∼75 min while the axon was positioned at the DREZ. These structures resemble the well-described filopodia observed during axon pathfinding (Chen et al., 2003; McCaig, 1989; Stanfield, 1966). When the axon was at the DREZ and filopodia were not present, growth cone actin instead organized into a dense concentration only observed for only 60-75 min in each axon (Fig. 6A).

Robust actin concentrations have been described in a variety of contexts (Hsia et al., 2003; Murphy and Courtneidge, 2011; Murphy et al., 2011; Santiago-Medina et al., 2015). Often, these concentrations project towards the basal lamina, which is present at the DREZ (Murphy and Courtneidge, 2011). Given descriptions of similar actin-based structures in other contexts, we hypothesized that this population was basally projecting. To test this, we sought to distinguish between these two populations by their three-dimensional morphologies by quantifying length in the *xy*-plane and *z*-plane. We generated *z*-plane images by digitally rotating stacks of individual time points and DRG. Using these measurements, we could identify distinct characteristics unique to each population. The filopodia-like population were longer in the *xy*-plane (5.06±0.31 µm), but only 0.567±0.02 µm in the *z*-plane (Fig. 6B-D; *n*=44 filopodia) consistent with previous descriptions of filopodia (Murphy and Courtneidge, 2011). The other population displayed the opposite characteristics: short in the *xy*-plane (0.956±0.05 µm; unpaired *t*-test comparing basally projecting and filopodia-like populations, *P*<0.001) and long in the *z*-plane at 1.04±0.04 µm (Fig. 6B-D; *n*=16 basally projecting clusters; unpaired *t*-test comparing basally projecting and filopodia-like populations, *P*<0.0001). These values are also consistent with descriptions of basally projecting actin populations (Murphy and Courtneidge, 2011; Nichols and Smith, 2019).

Although growth cone actin dynamics have been well-described, those in other neuronal domains are less understood. Therefore, we explored actin dynamics during axon initiation. In raw images, the axonal shaft appeared as a homogenous rod of F-actin, but DeSOS revealed unique populations. We first observed a population of actin within most of the axon shaft that was always present in the axon but was largely stable as it lagged the growth cone (Fig. 6E). With DeSOS, we also identified another distinct population in the axon shaft that localized to the base of extending neurites (Fig. 6E). Owing to this consistent concentration at the neurite base, we termed this population the stable base cluster (SBC). SBCs were present only during axon initiation and remained at the neurite base throughout neurite extension. To visualize SBC stability over time, we generated a kymograph of the extending axon, confirming a stable concentration of Lifeact-GFP at the base of the neurite throughout extension (Fig. 6E). We were also able to identify a third actin population that was present at this developmental time frame, the actin wave, the dynamics of which are described in detail below.

We next focused on the maturing axon, in which two distinct actin populations have been recently described: actin hotspots and actin trails (Ganguly et al., 2015). To test whether DeSOS can identify actin hotspots and trails *in vivo*, we created kymographs of mature axons. Because DRG neurons have multiple axonal domains, we investigated this possibility in each domain: the PNS-located central projection, the distal peripheral axon and the CNS-located central projection. In each domain, kymographs displayed a stable concentration of actin reminiscent of previous descriptions of actin hotspots (Fig. 6F). From each of these hotspots, we visualized anterograde and retrograde actin trails (Fig. 6F). In total, intravital DeSOS movies identified at least seven distinct actin populations in DRG axons.

### SBCs correlate with neurite extensions

**Fig. 7.**
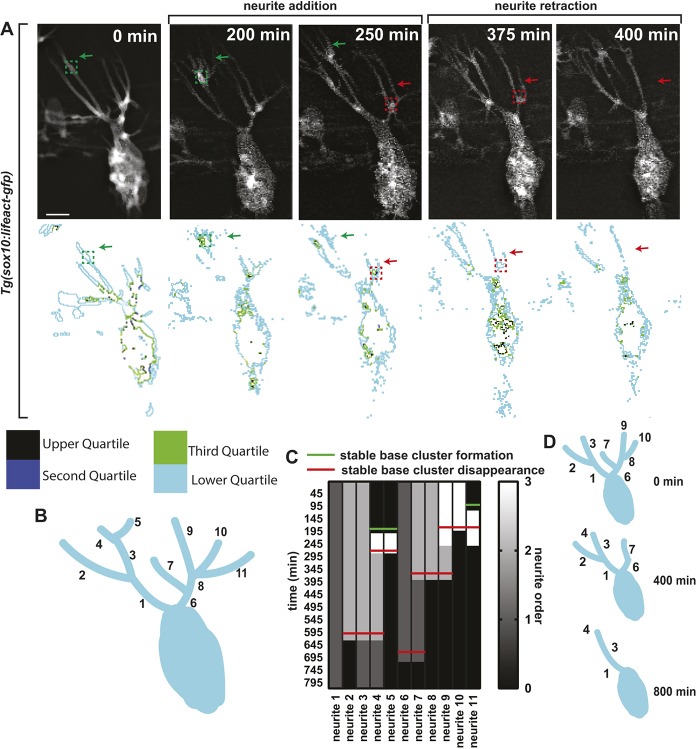
**Actin SBCs correlate with neurite extensions.** (A) Confocal *z*-projection DeSOS time-lapse images of a *Tg(sox10:gal4);* *Tg(uas:lifeact-GFP)* zebrafish showing axonal branching. Green boxes denote SBC actin populations throughout neurite addition. Green arrows denote neurite extension. Red boxes denote SBCs during neurite retraction. Red arrows denote neurite retraction. Composite outlines of each intensity quartile are shown. (B) Diagram of branching in DRG neurons that produce a pioneer axon. Neurites can be classified as primary (1,6), secondary (2,3,7,8) or tertiary (4,5,9,10,11). (C) Survival map of neurites scores dynamic neurite branching. Columns represent individual neurites; color denotes primary, secondary or tertiary classification over time. Green lines denote SBC formation. Red lines denote SBC disappearance. (D) Diagrams of neurite branching in DRG neurons over time (0, 400 and 800 min). Scale bar: 10 μm.

Of the actin populations that we visualized, little is known about SBCs. When SBCs are present, DRG neurons produce a single pioneer axon that projects to the DREZ. To dissect the formation of this single pioneer axon, we first investigated how neurite extensions create a single projection by tracking each individual neurite during axonal selection in DeSOS movies and identifying the neurite as primary (directly connected to the neuronal soma), secondary (branched from primary neurite), etc. Standard confocal movies do not provide enough spatial resolution to visualize all the axonal extensions that DeSOS detected. We reasoned that pioneer axons could be established by the extension of multiple primary neurites followed by one stabilizing while the rest retract. Alternatively, neurites could continuously add and subtract branches from existing neurites. In the movies, we observed that neurites extended from highly dynamic branch points (Fig. 7A). To score this phenomenon, we created a ‘survival map’ of neurites (Fig. 7B,C). Each column of the map represents an individual neurite over time, and the column shade at each time point represents whether the neurite was primary (dark gray), secondary (light gray), tertiary (white) or absent (black). This analysis showed that as branch points were eliminated, secondary and tertiary branches from the same tree were converted into primary and secondary branches, respectively (Fig. 7C,D). Further, tertiary neurites were the first to terminally stabilize into secondary, followed by the selection of secondary neurites into primary. Surprisingly, primary neurite selection was the final event in axon selection; this is inconsistent with a model in which the pioneer axon is generated from a single neurite that stabilizes before other neurites. Instead, these data support pioneer axon selection as a top-down scaffolding event driven by dynamic neurite and branch point remodeling.

To provide insight into the mechanism of top-down scaffolding of pioneer axon formation, we assayed the relationship of actin populations during this process. In DeSOS movies, we observed that SBCs were present at 92% of neurite branch points. Given the localization of SBCs to the neurite base, we hypothesized that branching and retraction events may correlate with SBCs. To test this, we recorded the time of SBC formation (green line) and disappearance (red line) in the survival map. This analysis revealed that SBC formation and disappearance preceded the initiation and retraction of neurites, suggesting that SBC formation could be essential for neurite extension.

If SBCs function in neurite extension and retraction, then their assembly or disassembly should predict neurite remodeling. We hypothesized that the presence or absence of SBCs determines the fate of its neurite. To test this, we measured the intensity of SBCs and the length of the associated neurites during neurite initiation. This analysis demonstrated that SBC intensity sharply increases 16±1.6 min prior to the extension of its associated neurite (Fig. S5A,B; *n*=10 SBC). Repeating this analysis with neurite retraction, we observe that the SBC decreased in intensity 29±4.4 min before neurite retraction (Fig. S5C,D; *n*=9 SBC). We never visualized branch retraction without the preceding destabilization of its SBC. Taken together, these data are consistent with the hypothesis that the presence of the SBC dictates neurite initiation and retraction.

### Actin waves between SBCs mediate neurite formation and retraction

**Fig. 8.**
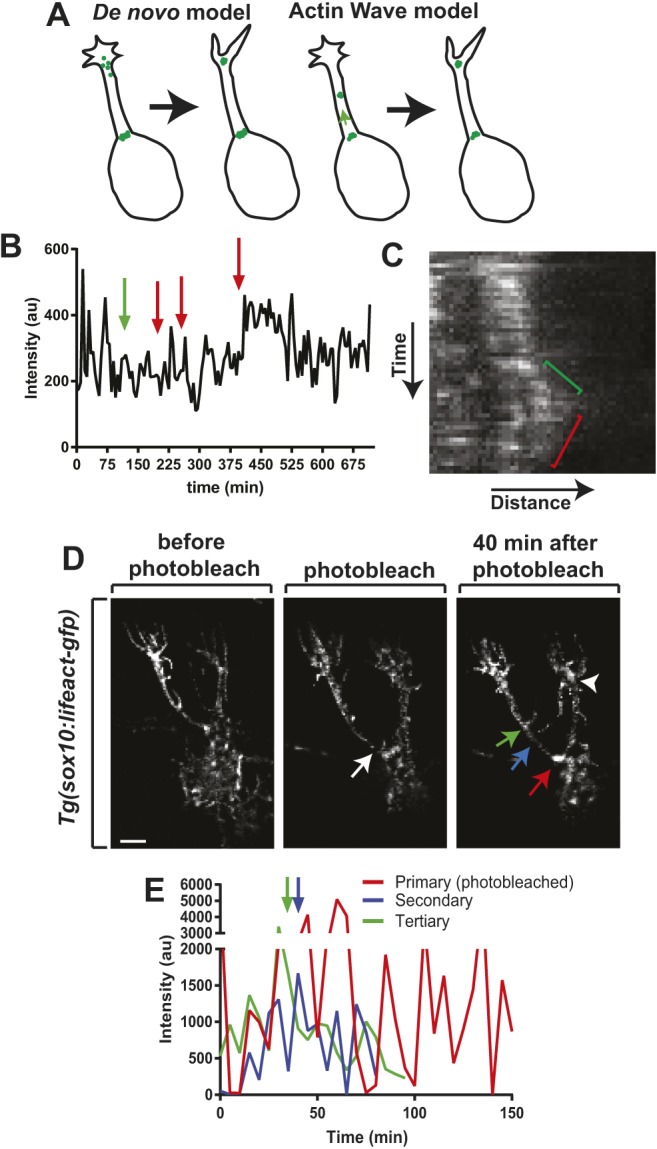
**Actin waves between SBCs mediate neurite formation and retraction.** (A) Two possible models of axon branching with SBC. One model predicts SBCs and neurites form *de novo* at a branch point. A second model proposes actin is mobilized from one SBC to the site of a new cluster and neurite. (B) Intensity of a single SBC over time. Green arrow denotes downstream neurite extension. Red arrows denote downstream neurite retraction. (C) Kymograph of SBC during the formation of a higher-order neurite. Green bracket denotes anterograde actin movement to a higher-order neurite. Red bracket denotes retrograde movement of actin to the SBC. (D) Confocal *z*-projection DeSOS images of a *Tg(sox10:gal4);* *Tg(uas:lifeact-GFP)* animal from a time-lapse movie before, during and after SBC photobleaching. White arrow denotes a photobleached SBC. Red arrow denotes a photobleached primary SBC, blue arrow denotes a secondary SBC, and green arrow denotes a tertiary SBC. White arrowhead denotes a second upstream SBC. (E) Intensity tracings of primary (photobleached), secondary and tertiary SBCs after photobleaching. Green arrow denotes initiation of tertiary neurite. Blue arrow denotes initiation of secondary neurite. An SBC was not initially detected for the secondary neurite. Scale bar: 10 µm.

To dissect this event further, we next hypothesized that primary or secondary SBCs may contribute to the formation of secondary or tertiary SBCs. To test this, we used MTrackJ to trace SBC intensity over time. These measurements showed that SBC intensity fluctuates, but when we overlaid the time points where downstream neurites (e.g. neurite 10 is downstream of the SBC of neurite 6) extended (green arrow) and retracted (red arrows), we consistently observed changes in SBC intensity (Fig. 8B). Specifically, SBC intensity decreased 14.8±3.9 min before a higher-order neurite initiated (*n*=6 SBC). Conversely, SBC intensity increased 30±10.7 min after higher-order neurite retraction (*n*=6 SBC). These data suggest that higher-order SBCs may be created from actin originally localized in lower-order clusters.

In this model of neurite selection and initiation, actin must be mobilized from primary SBCs to the site of the new cluster. Previous studies of actin dynamics in extending axons have shown that actin can be shuttled to branch points in ‘actin waves’ (Case and Waterman, 2011; Flynn et al., 2009; Inagaki and Katsuno, 2017). We hypothesized that actin waves are involved in the observed relationship between neurites and SBCs (Fig. 8A). Alternatively, SBCs and neurites could form *de novo* at a branch point (Fig. 8A). To dissect these possibilities, we created a kymograph of an SBC during the formation of higher-order neurites. This kymograph revealed that during higher-order neurite extension the SBC from the lower-order neurite retained a strong, albeit decreased, Lifeact-GFP intensity (Fig. 8C). We also observed anterograde and retrograde movement of actin from the lower-order base to the higher-order neurite. Anterograde movements led to decreased Lifeact-GFP at SBCs.

These data suggest that actin waves may be involved in moving actin from one SBC to another to precipitate neurite formation. We reasoned that if a lower-order SBC was photobleached, then we would visualize the formation of higher-order neurites without detecting an SBC. To test this, we photobleached a primary SBC and collected a time-lapse of neurite initiation. In doing so, we detected secondary neurite formation without visualizing its SBC (Fig. 8D). To confirm this, we traced SBC intensity of primary and secondary neurites. Even though the intensity of the primary SBC was restored following photobleaching, an SBC was not initially detected for the secondary neurite (Fig. 8E). Together, these data suggest that actin waves shuttle actin to higher-order SBCs and that SBCs continually receive new actin throughout their lifetime.

### Rac1 disrupts SBCs and pioneer axon selection

These data demonstrate that distinct actin populations coordinate during pioneer axon selection. We therefore sought to modify their stability by expressing photoactivatable Rac1 (PA-Rac1) in DRG neurons under the UAS promoter in *Tg(sox10:gal4); Tg(sox10:lifeact-GFP)* animals. We then collected DeSOS movies of these animals for 24 h from 48 hpf to 72 hpf (Movies 3, 4). PA-Rac1 was photoactivated by exposing the animal to 445 nm light every 5 min. We confirmed ectopic Rac1 activation by measuring the length of filopodia in DeSOS movies. Consistent with previous reports of Rac1-mediated filopodia stabilization, neurons with photoactivated Rac1 exhibited longer filopodia compared with non-photoactivated DRG (Fig. S6A; Student's *t*-test, *P*=0.0076; *n*=104 non-photoactivated, *n*=142 photoactivated) (Dumontier et al., 2000).

**Fig. 9.**
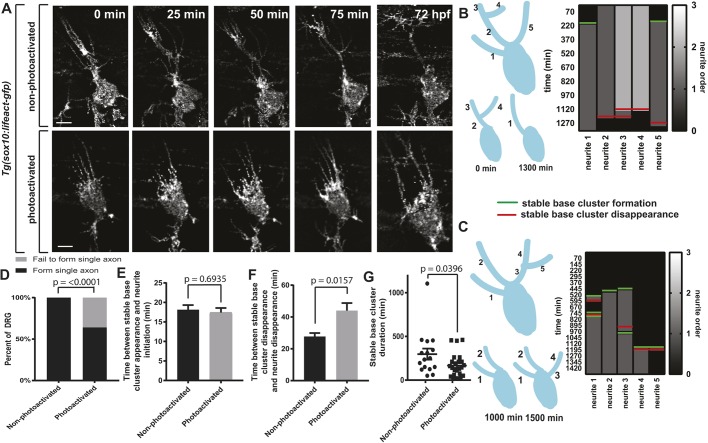
**Ectopic Rac1 disrupts SBCs and pioneer axon selection.** (A) Confocal *z*-projection time-lapse images of a *Tg(sox10:gal4);* *Tg(uas:lifeact-GFP)* animal during pioneer axon selection. *z*-stacks were taken every 5 min for 24 h starting at 48 hpf. (B) Left: Visualization of branching in non-photoactivated DRG (top) and further visualization of neurite branching in DRG neurons (0 and 1300 min) (bottom). Right: Survival map of neurites scoring dynamic neurite branching. (C) Left: Visualization of the branching in a photoactivated DRG (top) and further visualization of neurite dynamics in DRG neurons (1000 and 1500 min) (bottom). Right: Survival map of neurites scoring neurite branching. (D) Percentage of DRG that select a single axon. *n*=8 photoactivated DRG, *n*=11 non-photoactivated DRG. Fisher's exact test. (E) Time between SBC appearance and neurite initiation. *n*=16 non-photoactivated SBCs, *n*=29 photoactivated SBCs. (F) Time between SBC disappearance and neurite disappearance. *n*=18 non-photoactivated SBCs, *n*=31 photoactivated SBCs. (G) Quantifications of SBC duration. *n*=16 photoactivated SBCs, *n*=24 photoactivated SBCs. (E-G) Student's *t*-test. Scale bars: 10 μm.

We next hypothesized that ectopic Rac1 activation could disrupt pioneer axon selection via SBCs, neurite shafts, and/or actin waves. Further analysis of these movies showed that photoactivated neurons extended and retracted multiple primary neurites over short periods of time (Fig. 9A). This was confirmed in survival maps of pioneer axons with photoactivated Rac1, a phenotype not seen in non-photoactivated axons (Fig. 9B,C). This behavior was so strong that only 63% of DRG with photoactivated Rac1 selected a pioneer axon to extend to the DREZ (Fig. 9D; *n*=8 non-photoconverted, *n*=11 photoconverted; Fisher's exact test, *P*<0.0001).

We hypothesized that excessive primary neurite formation could be due to an altered ability to establish secondary SBCs and neurites via actin waves. To test this, we tracked SBC intensities over time and made kymographs of extending axons. We detected no changes in actin waves in photoactivated axons (Fig. S6B-E). We next hypothesized that altered SBC and neurite shaft dynamics could be responsible for the defects in pioneer axon formation. To test this, we quantified the time between SBC and neurite formations and detected no differences (Fig. 9E; *n*=16 non-photoactivated SBCs, *n*=29 photoactivated SBCs; Student's *t*-test, *P*=0.6935). We next measured the time between SBC disappearance and neurite retraction. In both photoactivated and non-photoactivated paradigms, neurite retraction always followed SBC disappearance. However, neurites with photoactivated Rac1 remained present after SBC disappearance longer than neurites expressing PA-Rac1 without photoactivation (Fig. 9F; *n*=18 non-photoactivated SBCs, *n*=31 photoactivated SBCs; Student's *t*-test, *P*=0.0157). The simplest explanation for these data is that Rac1 stabilizes extending neurite branches, presumably by favoring actin localization in the neurite shafts, consequently decreasing actin levels in SBCs. To test this, we measured SBC duration. Photoactivated axons produced SBCs that were present for 166.9±27.25 min, compared with 297.8±63.46 min for SBCs in non-photoactivated axons (Fig. 9G; *n*=16 non-photoactivated SBCs, *n*=24 photoactivated SBCs; Student's *t*-test, *P*=0.0396). Taken together, these data suggest that Rac1 favors actin localization in extending neurites over SBCs. Without a steady SBC, the axon cannot successfully extend to targets.

## DISCUSSION

Here, we introduce a highly accessible super-resolution technique, DeSOS, that can be employed in intravital and live-animal imaging. We demonstrate that super-resolution can be obtained using two images taken at low excitation powers from conventional microscopes. We show the utility of this technique in both fixed and living tissue and provide an open-access application for the generation of DeSOS images. DeSOS can be used to obtain super-resolution time-lapse movies shown here by visualizing dynamic actin populations in nascent DRG neurons. Together, our data introduce DeSOS as an intravital super-resolution technique that can be used to construct super-resolution time-lapse movies that will have a robust impact in studying cells in their native physiological environments.

### DeSOS visualizes cells in their native environments

The development of super-resolution imaging techniques has increased our understanding of subcellular structure and organization (Ganguly et al., 2015; Liu et al., 2018; Tønnesen et al., 2018). However, most optical super-resolution techniques (SIM, STORM, etc.) require thin slices of tissue, effectively precluding their use in intact or living animals. In this way, super-resolution techniques make it fundamentally difficult to image cells in their native physiological environments, an important consideration as interactions with other cells and its microenvironment influence cellular assembly and organization (Di Martino et al., 2016; Kang et al., 2003; Pan and Chan, 2017). Recently, expansion microscopy, which physically expands the tissue sample, has been proposed to circumvent this limitation (Chen et al., 2015; Chozinski et al., 2016; Freifeld et al., 2017). However, this technique requires cross-linking fluorophores to a polymer network, preventing use in living animals (Chozinski et al., 2016; Freifeld et al., 2017). DeSOS overcomes both disadvantages, and we provide an open-access application that can be used to easily obtain DeSOS images, making DeSOS highly accessible to scientists across disciplines.

Intravital super-resolution has been achieved previously with STED with impressive resolution of ∼1 nm (Balzarotti et al., 2017). STED requires extensive instrumentation, including an additional laser to induce stimulated emission of fluorophores, a vortex phase plate or a spatial light modulator to generate depletion (i.e. donut-shape) patterns, a circuitry to synchronize the onset of the two laser beams and the detector, and additional optical alignment and calibration procedures (Blom and Widengren, 2017). STED usually demands high excitation power to quench the fluorophores surrounding the focal point through stimulated emission. Therefore, STED was long considered incompatible with long-term and live-cell imaging owing to photobleaching and phototoxicity caused by the high excitation power (Vicidomini et al., 2018); in comparison, we successfully demonstrate long-term (24 h), intravital, DeSOS imaging using low excitation power. STED can also be limited in its imaging depth, as scattering and aberrations can break the co-alignment of the two beams used for STED imaging. DeSOS, on the other hand, has a deep penetration depth as scattering does not adversely degrade image quality other than reducing the SNR. Despite the differences, STED remains the gold standard of super-resolution and outperforms DeSOS in many ways, especially its resolution of ∼1 nm (Balzarotti et al., 2017). DeSOS provides a moderate but more accessible super-resolution solution that can perform long-term intravital imaging at no additional cost.

Similar in accessibility to DeSOS, SRRF provides super-resolution images with no additional hardware and requires acquisition of >100 frames that are computationally processed to generate a single super-resolution image (Gustafsson et al., 2016). DeSOS can be easily applied to volume stacks whereas volumetric imaging with SRRF does not provide improvement in *z*-resolution like DeSOS (Gustafsson et al., 2016; Zhang et al., 2018). Our data also suggest that DeSOS retains quantitative information that SRRF does not. However, and importantly, SRRF and DeSOS are theoretically independent, allowing them to be potentially combined to improve overall resolution. Similarly, as DeSOS is widely accessible to confocal and two-photon systems it can also theoretically be combined with other imaging modalities to fit the desired experiment.

### Super-resolution movies image cellular processes in living animals

Here, we use DeSOS movies to visualize the dynamic actin organization in nascent DRG neurons as they produce a pioneer axon and mature. The mechanisms by which neurons produce striking polarity through the creation of axons and dendrites has long been of interest. Current models of axonal selection emphasize a competition between neurites for cellular resources or the presence of an intracellular sensor in one neurite that downregulates growth in neighboring neurites (Inagaki et al., 2011; Toriyama et al., 2010; Winans et al., 2016). These hypotheses have been resolved largely in cell culture systems. DeSOS allows us to test such hypotheses in living animals and visualize the selection of the pioneer axon in DRG neurons. Our movies indicate that neurite selection, at least in DRG, occurs in a top-down process whereby distal targeting is selected before the more proximal neurite segments. These data suggest that pioneer axon selection occurs in multiple stages, first at local levels within individual primary neurites and later between primary neurites themselves. Further, our pioneer axon selection data in living animals highlights the importance of SBCs in this process. Future studies will now be able to take advantage of DeSOS’ compatibility with intravital imaging to test additional hypotheses in cells' native environments.

## MATERIALS AND METHODS

### Experimental model and subject details

All animal studies were approved by the University of Notre Dame Institutional Animal Care and Use Committee. The zebrafish and mouse strains used in this study are listed in supplementary Materials and Methods. All zebrafish were raised at 28°C in egg water in constant darkness and staged by hpf or dpf.

#### *In vivo* imaging with spinning disk confocal microscopy of zebrafish

Zebrafish were imaged as previously described (Nichols and Smith, 2019) on a confocal microscope. Further details on fish imaging and the microscope used for this study can be found in supplementary Materials and Methods. Images for SRRF were collected as 100 consecutive frames (Gustafsson et al., 2016). SRRF images of individual *z*-planes were generated using NanoJ software for ImageJ. SRRF images of individual *z*-planes were then collapsed into a *z*-projection.

#### Intravital microscopy of mice

Mice were subjected to skull thinning and imaged on a two-photon microscope. Further details are noted in supplementary Materials and Methods.

#### DeSOS microscopy

The fluorescent images obtained with the spinning disk confocal microscope were processed with DeSOS microscopy, which consisted of two operations: blind deconvolution and SOS. The details of each step can be found in supplementary Materials and Methods. The open access DeSOS program is hosted at: https://dx.doi.org/doi:10.7274/r0-5hhg-5578.

#### Simulation

The conventional confocal PSFs were simulated based on the intensity distribution in the focal region of an aberration-free lens of circular aperture (Sheppard and Gu, 1990; Sheppard and Matthews, 1987) and the steady-state solution of a two-level saturable fluorophore model (Zhang et al., 2018). Further details of the object simulation are given in supplementary Materials and Methods.

#### Bead imaging

Bead imaging experiments were performed on the spinning disk confocal microscope. A prepared slide mounted with fluorescent microspheres of different sizes (Invitrogen, T14792) was used for the imaging. We chose the 500 nm diameter beads with a concentration of 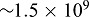 particles/ml. The excitation wavelength was 488 nm; the stack dimension was 1024×1024×32, with a pixel width of 206 nm and a slice depth of 1 μm. The image for the first step was taken with a power of 59.1 μW and the second step was taken with 72.7 μW. The raw image stacks were imported to AutoQuant Blind for deconvolution. The raw and deconvolved images were then imported to Matlab for SOS and DeSOS processing. A ROI of a single bead was chosen and the images were cropped to this ROI to demonstrate the resolution of each imaging modality. The lateral and axial profiles of the bead were fitted to a Gaussian function and the corresponding FWHMs were measured.

#### PA-Rac1 expression

The *tol2-nxr UAS-PA Rac1-mcherry* plasmid was stored at a working concentration of 75 ng/µl. An injection solution was prepared in water with 12 ng/µl of the plasmid and 25 ng/µl of a plasmid encoding *transposase*. This solution was injected into *Tg(sox10:gal4); Tg(uas:lifeact-GFP)* embryos at the single cell stage. At 48 hpf, PA-Rac1-mcherry^+^ DRG were identified using confocal microscopy. These DRG were time-lapse imaged and processed for DeSOS as described in supplementary Materials and Methods. The *tol2-nxr UAS-PA Rac1-mcherry* plasmid was a gift from Anna Huttenlocher (Addgene plasmid #41878).

#### DeSOS movies

DeSOS movies were generated in collaboration with the Center for Research Computing at the University of Notre Dame. Time-lapse images were taken at excitation powers 59.1 µW and 86.3 µW every 5 min for 24 h. Individual images at each time point were deconvolved using the Autoquant Blind algorithm. These deconvolved images were uploaded to the Center for Research Computing's high-performance computing servers using the Cyberduck app. The SOS algorithm was then remotely applied on the deconvolved time-lapse images. Following SOS processing, the images were locally re-downloaded via Cyberduck and compiled into a DeSOS movie.

### Quantification and statistical analysis

Slidebook software was used to create maximum intensity projections for all images and movies. Quantification of all data was carried out using ImageJ. All graphical data represents the mean and s.e.m. unless otherwise noted. All statistical analyses were completed using GraphPad Prism software. A detailed explanation of individual quantifications can be found in supplementary Materials and Methods and Table S1.

## Supplementary Material

Supplementary information

## References

[DEV171512C1] Balzarotti F., Eilers Y., Gwosch K. C., Gynnå A. H., Westphal V., Stefani F. D., Elf J. and Hell S. W. (2017). Nanometer resolution imaging and tracking of fluorescent molecules with minimal photon fluxes. *Science* 355, 606-612. 10.1126/science.aak9913 28008086

[DEV171512C2] Betzig E., Patterson G. H., Sougrat R., Lindwasser O. W., Olenych S., Bonifacino J. S., Davidson M. W., Lippincott-Schwartz J. and Hess H. F. (2006). Imaging intracellular fluorescent proteins at nanometer resolution. *Science* 313, 1642-1645. 10.1126/science.1127344 16902090

[DEV171512C3] Blom H. and Widengren J. (2017). Stimulated emission depletion microscopy. *Chem. Rev.* 117, 7377-7427. 10.1021/acs.chemrev.6b00653 28262022

[DEV171512C4] Case L. B. and Waterman C. M. (2011). Adhesive F-Actin waves: a novel integrin-mediated adhesion complex coupled to ventral actin polymerization. *PLoS One* 6, e26631 10.1371/journal.pone.0026631 22069459 PMC3206032

[DEV171512C5] Chen N., Furuya S., Doi H., Hashimoto Y., Kudo Y. and Higashi H. (2003). Ganglioside/calmodulin kinase II signal inducing cdc42-mediated neuronal actin reorganization. *Neuroscience* 120, 163-176. 10.1016/S0306-4522(03)00259-8 12849750

[DEV171512C6] Chen F., Tillberg P. W. and Boyden E. S. (2015). Expansion microscopy. *Science* 347, 543-548. 10.1126/science.1260088 25592419 PMC4312537

[DEV171512C7] Chozinski T. J., Halpern A. R., Okawa H., Kim H.-J., Tremel G. J., Wong R. O. L. and Vaughan J. C. (2016). Expansion microscopy with conventional antibodies and fluorescent proteins. *Nat. Methods* 13, 485-488. 10.1038/nmeth.3833 27064647 PMC4929147

[DEV171512C8] Cole R. W., Jinadasa T. and Brown C. M. (2011). Measuring and interpreting point spread functions to determine confocal microscope resolution and ensure quality control. *Nat. Protoc.* 6, 1929-1941. 10.1038/nprot.2011.407 22082987

[DEV171512C9] Di Martino J., Henriet E., Ezzoukhry Z., Goetz J. G., Moreau V. and Saltel F. (2016). The microenvironment controls invadosome plasticity. *J. Cell Sci.* 129, 1759-1768. 10.1242/jcs.182329 27029343

[DEV171512C10] Dumontier M., Höcht P., Mintert U. and Faix J. (2000). Rac1 GTPases control filopodia formation, cell motility, endocytosis, cytokinesis and development in Dictyostelium. *J. Cell Sci.* 113, 2253-2265. 10825297 10.1242/jcs.113.12.2253

[DEV171512C11] Flynn K. C., Pak C. W., Shaw A. E., Bradke F. and Bamburg J. R. (2009). Growth cone-like waves transport actin and promote axonogenesis and neurite branching. *Dev. Neurobiol.* 69, 761-779. 10.1002/dneu.20734 19513994 PMC2845293

[DEV171512C12] Freifeld L., Odstrcil I., Förster D., Ramirez A., Gagnon J. A., Randlett O., Costa E. K., Asano S., Celiker O. T., Gao R. et al. (2017). Expansion microscopy of zebrafish for neuroscience and developmental biology studies. *Proc. Natl. Acad. Sci. USA* 114, E10799-E10808. 10.1073/pnas.1706281114 29162696 PMC5740639

[DEV171512C13] Ganguly A., Tang Y., Wang L., Ladt K., Loi J., Dargent B., Leterrier C. and Roy S. (2015). A dynamic formin-dependent deep F-actin network in axons. *J. Cell Biol.* 210, 401-417. 10.1083/jcb.201506110 26216902 PMC4523607

[DEV171512C14] Gustafsson M. G. L. (2005). Nonlinear structured-illumination microscopy: Wide-field fluorescence imaging with theoretically unlimited resolution. *Proc. Natl. Acad. Sci. USA* 102, 13081-13086. 10.1073/pnas.0406877102 16141335 PMC1201569

[DEV171512C15] Gustafsson N., Culley S., Ashdown G., Owen D. M., Pereira P. M. and Henriques R. (2016). Fast live-cell conventional fluorophore nanoscopy with ImageJ through super-resolution radial fluctuations. *Nat. Commun.* 7, 12471 10.1038/ncomms12471 27514992 PMC4990649

[DEV171512C16] Heap L. A., Goh C. C., Kassahn K. S. and Scott E. K. (2013). Cerebellar output in zebrafish: an analysis of spatial patterns and topography in eurydendroid cell projections. *Front. Neural Circuits* 7, 53. 23554587 10.3389/fncir.2013.00053 PMC3612595

[DEV171512C17] Helker C. S. M., Schuermann A., Karpanen T., Zeuschner D., Belting H.-G., Affolter M., Schulte-Merker S. and Herzog W. (2013). The zebrafish common cardinal veins develop by a novel mechanism: lumen ensheathment. *Development* 140, 2776-2786. 10.1242/dev.091876 23698350

[DEV171512C18] Hell S. W. and Wichmann J. (1994). Breaking the diffraction resolution limit by stimulated emission: stimulated-emission-depletion fluorescence microscopy. *Opt. Lett.* 19, 780-782. 10.1364/OL.19.000780 19844443

[DEV171512C19] Hines J. H., Ravanelli A. M., Schwindt R., Scott E. K. and Appel B. (2015). Neuronal activity biases axon selection for myelination in vivo. *Nat. Neurosci.* 18, 683-689. 10.1038/nn.3992 25849987 PMC4414883

[DEV171512C20] Holmes T. J., Biggs D. and Abu-Tarif A. (2006). Blind deconvolution. In *Handbook of Biological Confocal Microscopy* (ed. J. B. Pawley), pp. 468-487. Boston, MA: Springer US.

[DEV171512C21] Hsia D. A., Mitra S. K., Hauck C. R., Streblow D. N., Nelson J. A., Ilic D., Huang S., Li E., Nemerow G. R., Leng J. et al. (2003). Differential regulation of cell motility and invasion by FAK. *J. Cell Biol.* 160, 753-767. 10.1083/jcb.200212114 12615911 PMC2173366

[DEV171512C22] Inagaki N. and Katsuno H. (2017). Actin waves: origin of cell polarization and migration? *Trends Cell Biol.* 27, 515-526. 10.1016/j.tcb.2017.02.003 28283221

[DEV171512C23] Inagaki N., Toriyama M. and Sakumura Y. (2011). Systems biology of symmetry breaking during neuronal polarity formation. *Dev. Neurobiol.* 71, 584-593. 10.1002/dneu.20837 21557507

[DEV171512C24] Kang Y., Siegel P. M., Shu W., Drobnjak M., Kakonen S. M., Cordón-Cardo C., Guise T. A. and Massagué J. (2003). A multigenic program mediating breast cancer metastasis to bone. *Cancer Cell* 3, 537-549. 10.1016/S1535-6108(03)00132-6 12842083

[DEV171512C25] Kucenas S., Takada N., Park H.-C., Woodruff E., Broadie K. and Appel B. (2008). CNS-derived glia ensheath peripheral nerves and mediate motor root development. *Nat. Neurosci.* 11, 143-151. 10.1038/nn2025 18176560 PMC2657597

[DEV171512C26] Liu T. L., Upadhyayula S., Milkie D. E., Singh V., Wang K., Swinburne I. A., Mosaliganti K. R., Collins Z. M., Hiscock T. W., Shea J. et al. (2018). Observing the cell in its native state: imaging subcellular dynamics in multicellular organisms. *Science* 360, eaaq1392 10.1126/science.aaq1392 29674564 PMC6040645

[DEV171512C27] MacDonald L., Baldini G. and Storrie B. (2015). Does super-resolution fluorescence microscopy obsolete previous microscopic approaches to protein co-localization? In *Methods in Molecular Biology* (ed. Tang B. L.), pp. 255-275. New York, NY: Springer New York. 10.1007/978-1-4939-2309-0_19 PMC4389218 25702123

[DEV171512C28] McCaig C. D. (1989). Nerve growth in the absence of growth cone filopodia and the effects of a small applied electric field. *J. Cell Sci.* 93, 715-721. 2606944 10.1242/jcs.93.4.715

[DEV171512C29] McNally J. G., Karpova T., Cooper J. and Conchello J. A. (1999). Three-dimensional imaging by deconvolution microscopy. *Methods* 19, 373-385. 10.1006/meth.1999.0873 10579932

[DEV171512C30] Murphy D. A. and Courtneidge S. A. (2011). The ‘ins’ and ‘outs’ of podosomes and invadopodia: characteristics, formation and function. *Nat. Rev. Mol. Cell Biol.* 12, 413-426. 10.1038/nrm3141 21697900 PMC3423958

[DEV171512C31] Murphy D. A., Diaz B., Bromann P. A., Tsai J. H., Kawakami Y., Maurer J., Stewart R. A., Izpisúa-Belmonte J. C. and Courtneidge S. A. (2011). A Src-Tks5 pathway is required for neural crest cell migration during embryonic development. *PLoS One* 6, e22499 10.1371/journal.pone.0022499 21799874 PMC3143166

[DEV171512C32] Nichols E. L. and Smith C. J. (2019). Pioneer axons employ Cajal's battering ram to enter the spinal cord. *Nat. Commun*. 10, 562 10.1038/s41467-019-08421-9 30718484 PMC6362287

[DEV171512C33] Pan S. and Chan J. R. (2017). Regulation and dysregulation of axon infrastructure by myelinating glia. *J. Cell Biol.* 216, 3903-3916. 10.1083/jcb.201702150 29114067 PMC5716274

[DEV171512C34] Rust M. J., Bates M. and Zhuang X. (2006). Sub-diffraction-limit imaging by stochastic optical reconstruction microscopy (STORM). *Nat. Methods* 3, 793-796. 10.1038/nmeth929 16896339 PMC2700296

[DEV171512C35] Sage D., Donati L., Soulez F., Fortun D., Schmit G., Seitz A., Guiet R., Vonesch C. and Unser M. (2017). DeconvolutionLab2: an open-source software for deconvolution microscopy. *Methods* 115, 28-41. 10.1016/j.ymeth.2016.12.015 28057586

[DEV171512C36] Santiago-Medina M., Gregus K. A., Nichol R. H., O'Toole S. M. and Gomez T. M. (2015). Regulation of ECM degradation and axon guidance by growth cone invadosomes. *Development* 142, 486-496. 10.1242/dev.108266 25564649 PMC4302990

[DEV171512C37] Sheppard C. and Gu M. (1990). Image formation in two-photon fluorescence microscopy. *Optik (Stuttg).* 86, 104-106.

[DEV171512C38] Sheppard G. J. R. and Matthews H. J. (1987). Imaging in high-aperture optical systems. *J. Opt. Soc. Am. A* 4, 1354 10.1364/JOSAA.4.001354

[DEV171512C39] Smith C. J., Wheeler M. A., Marjoram L., Bagnat M., Deppmann C. D. and Kucenas S. (2017). TNFa/TNFR2 signaling is required for glial ensheathment at the dorsal root entry zone. *PLoS Genet.* 13, e1006712 10.1371/journal.pgen.1006712 28379965 PMC5397050

[DEV171512C40] Stanfield A. B. (1966). Cells with filopodia cultured from human synovialis. *Anat. Rec.* 154, 73-79. 10.1002/ar.1091540106 4162459

[DEV171512C41] Strauss M. P., Liew A. T. F., Turnbull L., Whitchurch C. B., Monahan L. G. and Harry E. J. (2012). 3D-SIM super resolution microscopy reveals a bead-like arrangement for FtsZ and the division machinery: implications for triggering cytokinesis. *PLoS Biol.* 10, e1001389 10.1371/journal.pbio.1001389 22984350 PMC3439403

[DEV171512C42] Tønnesen J., Inavalli V. V. G. K. and Valentin Nä Gerl U. V. (2018). Super-resolution imaging of the extracellular space in living brain tissue. *Cell* 172, 1108-1121. 10.1016/j.cell.2018.02.007 29474910

[DEV171512C43] Toriyama M., Sakumura Y., Shimada T., Ishii S. and Inagaki N. (2010). A diffusion-based neurite length-sensing mechanism involved in neuronal symmetry breaking. *Mol. Syst. Biol.* 6, 394 10.1038/msb.2010.51 20664640 PMC2925530

[DEV171512C44] Vicidomini G., Bianchini P. and Diaspro A. (2018). STED super-resolved microscopy. *Nat. Methods* 15, 173-182. 10.1038/nmeth.4593 29377014

[DEV171512C45] Winans A. M., Collins S. R. and Meyer T. (2016). Waves of actin and microtubule polymerization drive microtubule-based transport and neurite growth before single axon formation. *Elife* 5, e12387 10.7554/eLife.12387 26836307 PMC4805541

[DEV171512C46] Zhang Y., Nallathamby P. D., Vigil G. D., Khan A. A., Mason D. E., Boerckel J. D., Roeder R. K. and Howard S. S. (2018). Super-resolution fluorescence microscopy by stepwise optical saturation. *Biomed. Opt. Express* 9, 1631-1629. 10.1364/BOE.9.001613 PMC5905910 29675306

