# Supplementary material for: Generating intravital super-resolution movies with conventional microscopy reveals actin dynamics that construct pioneer axons
Source: Development. 2019 Mar 8;146(5):dev171512. doi: 10.1242/dev.171512 (PMC6432666; doi:10.1242/dev.171512)
Supplement: Supplementary information [file develop-146-171512-s1.pdf]

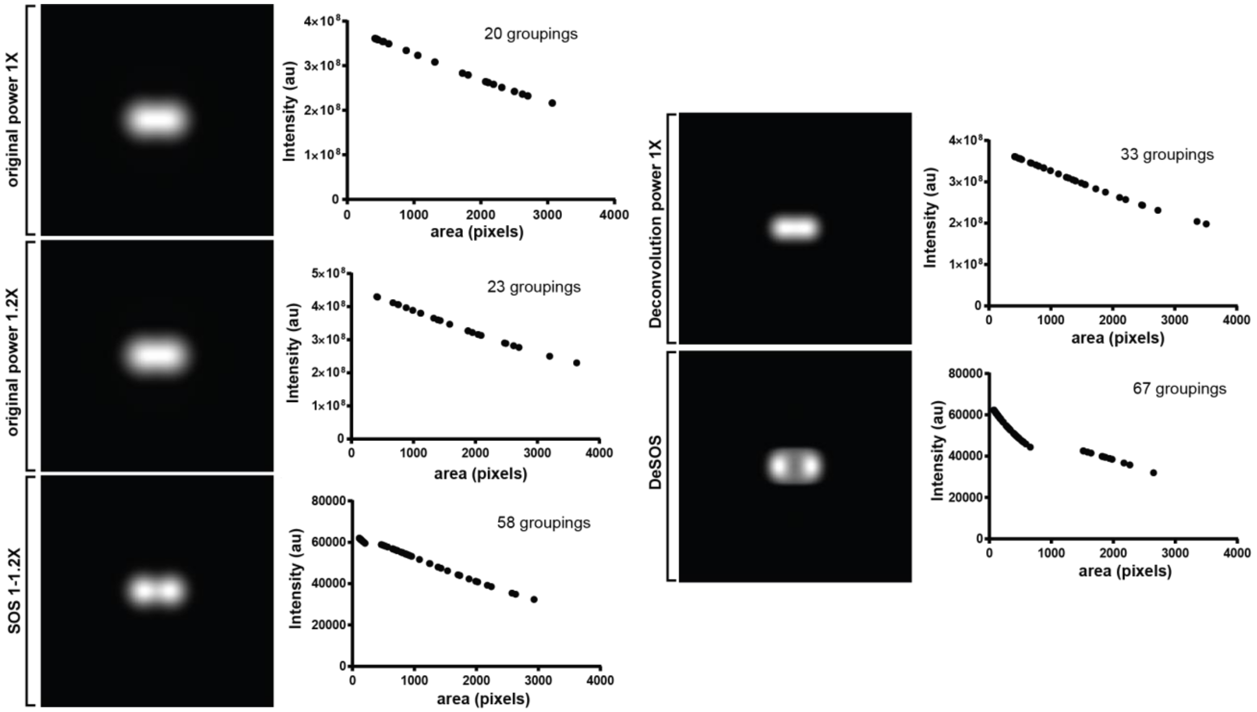

Figure S1. Calibration of pixel-grouping as a measure of image resolution, related to Figure 3. Z-projection simulated images of two fluorescent objects separated by 0.3X of the acquisition wavelength. Adjacent pixels that had intensity values within a 10% range of the maximum pixel intensity grouped and the number of those groupings counted for each image and represented by their areas and mean intensities. Increased numbers of pixel groupings is strongly associated with increasing resolution of SOS and DeSOS images.

Development • Supplementary information

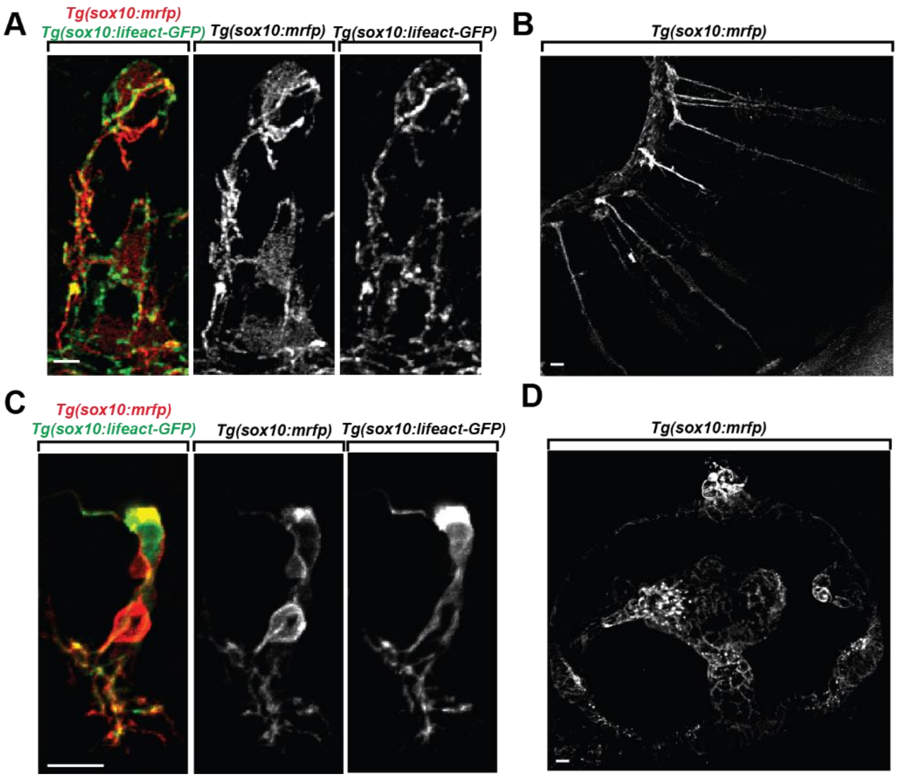

Figure S2. Utility of DeSOS technique in distinct anatomical regions and fluorophores, related to Figures 3 and 4. (A) Confocal z-projection DeSOS images at 48 hpf of an oligodendrocyte progenitor in a fixed *Tg(sox10:mrfp)*; *Tg(sox10:lifeact-gfp)* animal. (B) Confocal z-projection DeSOS image at 48 hpf of the retina in a fixed *Tg(sox10:mrfp)* animal. (C) Confocal z-projection DeSOS images at 48 hpf of an oligodendrocyte progenitor in a live *Tg(sox10:mrfp)*; *Tg(sox10:lifeact-gfp)* animal. (D) Confocal z-projection DeSOS image at 48 hpf of the otic vesicle in a live *Tg(sox10:mrfp)* animal. Scale bar is 10 μm

Development • Supplementary information

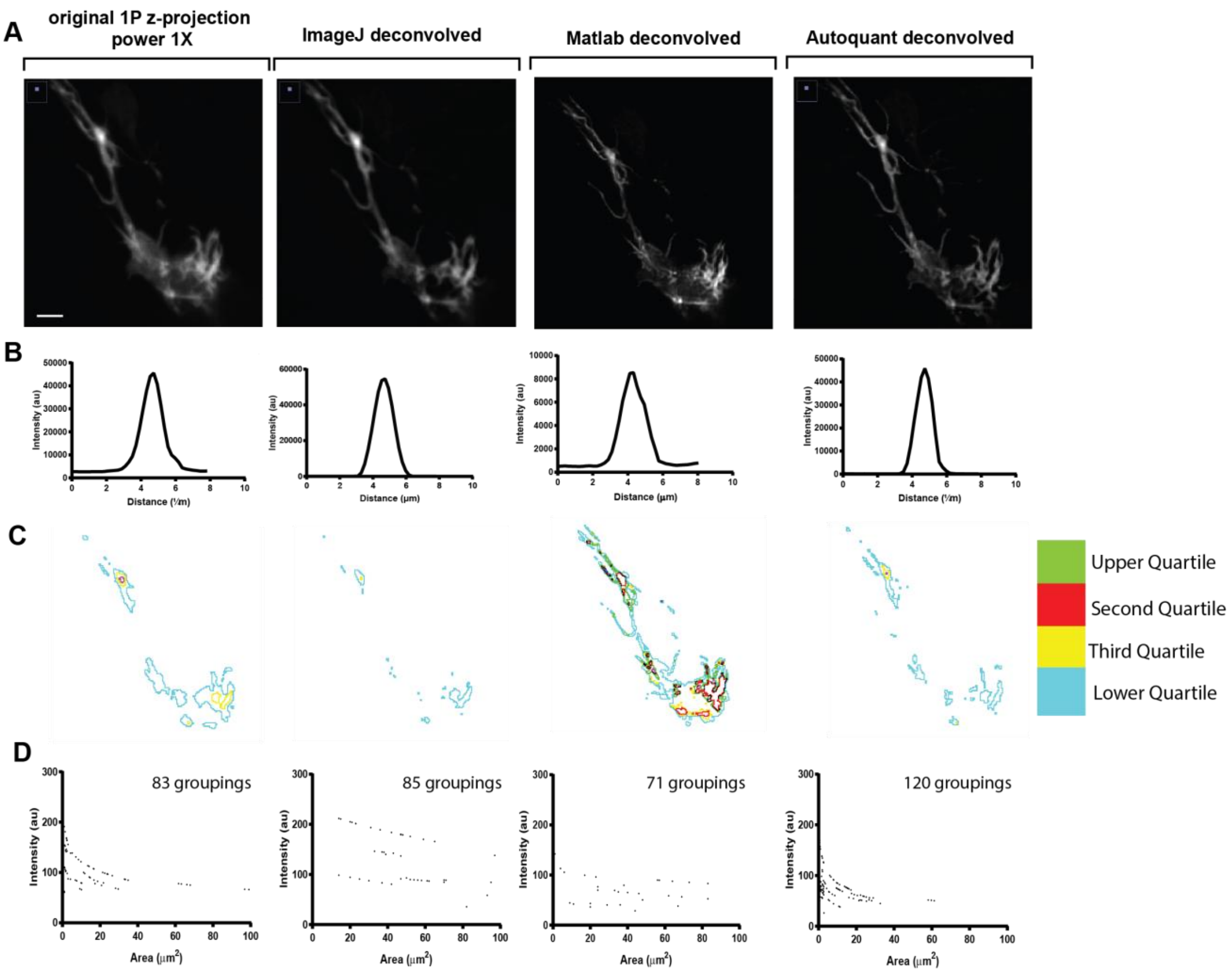

Figure S3. Distinct deconvolution techniques improve signal-to-noise ratio, related to Figure 3. (A) Confocal z-projection images of fixed tissue at 48 hpf of *Tg(sox10:gal4)*; *Tg(uas:lifeact-GFP)* zebrafish. Image was taken at powers 1X (1.011 mW) and 1.014X (1.025 mW) and deconvolved using the ImageJ plugin DeconvolutionLab2, Matlab, and Autoquant Blind. (B) Graph of pixel intensity across a designated line through a (ROI) in the neuron with concentrated Lifeact-GFP for each image in (A). (C) Pixels in each image subdivided into quartiles by intensity to visualize distinct populations of pixels. Composite outlines of each quartile are shown. (D) Adjacent pixels that had intensity values within a 10% range of the maximum pixel intensity grouped and the number of groupings for each image in (A) and represented by their areas and mean intensities. Scale bar is 10 μm.

Development • Supplementary information

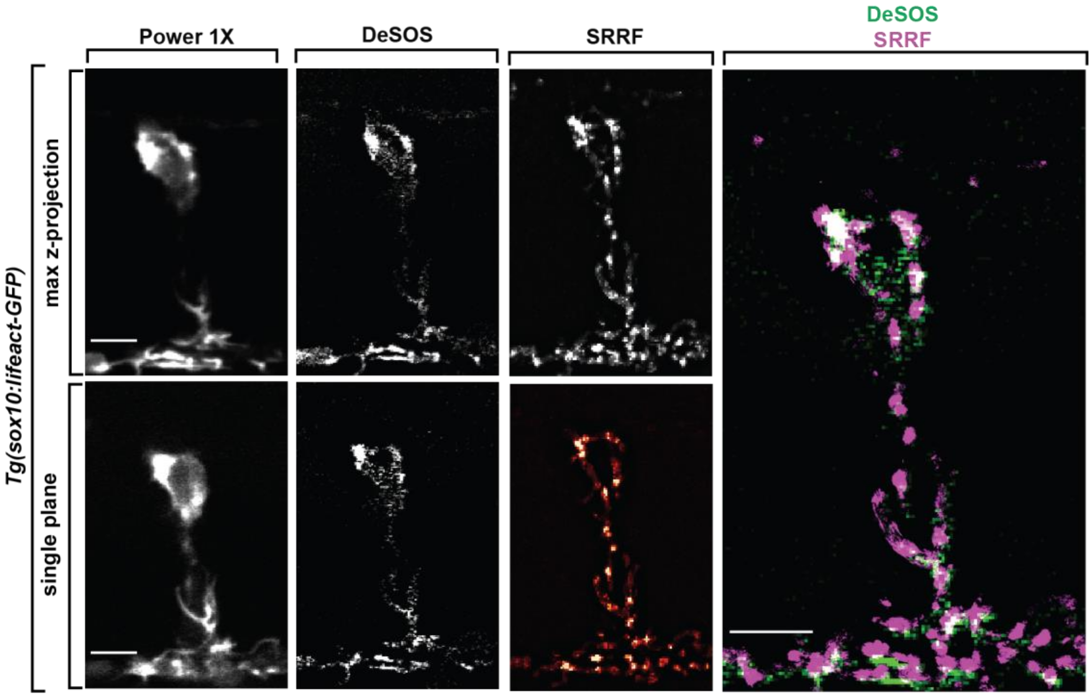

Figure S4. Comparison of DeSOS and SRRF, related to Figure 4. (A) Confocal z-projection and single plane images of live tissue at 48 hpf of *Tg(sox10:gal4)*; *Tg(uas:lifeact-GFP)* zebrafish. First, images were taken at laser power 1X (1.011 mW) and 1.014X (1.025 mW) for generation of a DeSOS image. Immediately after, 100 consecutive frames were taken for SRRF processing. The raw power 1X, DeSOS, and SRRF images are shown. An overlap of the maximum z-protection DeSOS (green) and SRRF (magenta) images provide a direct comparison of the two super-resolution images. Scale bar is 10 μm

Development • Supplementary information

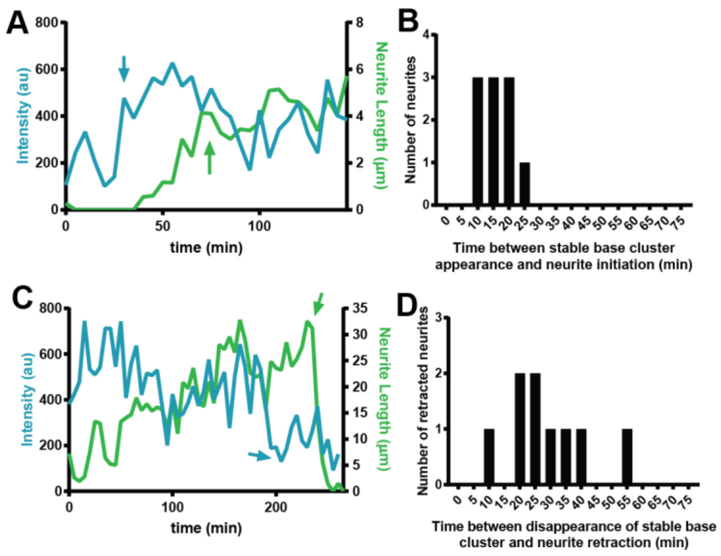

Figure S5. Neurite branching and retraction events correlate with stable base clusters, related to Figure 7. (A) Graph measuring the intensity of the stable base cluster (blue) and the length of its associated neurite (green) during neurite initiation. Blue arrow denotes an increase in intensity of the stable base cluster directly prior to the extension of its associated neurite (green arrow). (B) Histogram of the time between stable base cluster appearance and neurite initiation (n = 10 neurites). (C) Graph measuring the intensity of the stable base cluster (blue) and the length of its associated neurite (green) during neurite retraction. Blue arrow denotes a decrease in intensity of the stable base cluster directly prior to the retraction of its associated neurite (green arrow). (D) Histogram of the time between stable base cluster disappearance and neurite retraction (n = 9 neurites).

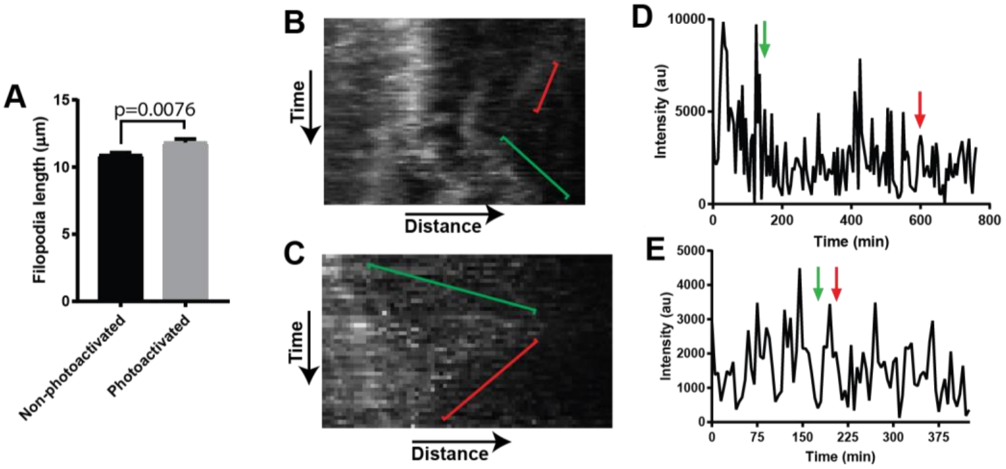

Figure S6: Axon selection in non-photoactivated and photoactivated DRG, related to Figure 9. (A) Graph of filopodia length in the xy-plane for non-photoactivated and photoactivated DRG (n = 104 non-photoactivated, n = 142 photoactivated) (B) Kymograph of actin hotspot and actin trails in a non-photoactivated DRG axon. Red bracket denotes an actin trail moving in a retrograde direction. Green bracket denotes an actin trail moving in an anterograde direction. (C) Kymograph of actin hotspot and actin trails in a photoactivated DRG axon. Green bracket denotes an actin trail moving in an anterograde direction. Red bracket denotes an actin trail moving in a retrograde direction. (D) Graph of Lifeact-GFP intensity of a stable base cluster over time in a non-photoactivated DRG axon. Green arrow denotes the initiation of a downstream neurite. Red arrow denotes the retraction of a downstream neurite. (E) Graph of Lifeact-GFP intensity of a stable base cluster over time in a photoactivated DRG axon. Green arrow denotes the initiation of a downstream neurite. Red arrow denotes the retraction of a downstream neurite. SEM bars are shown (A).

Development • Supplementary information

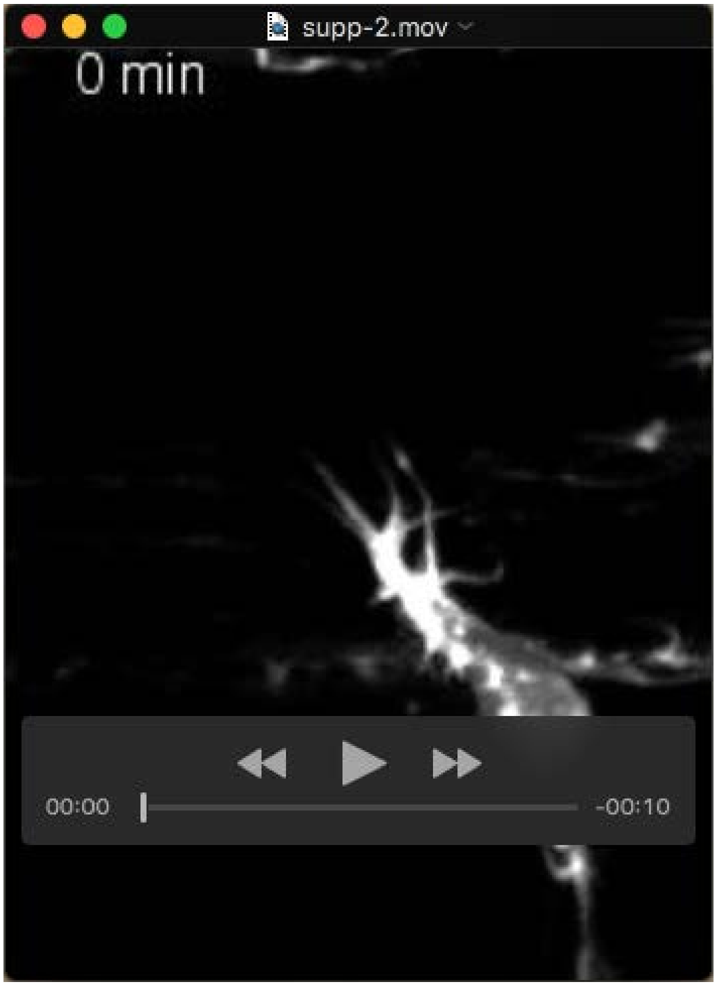

Movie 1. Power 1X time-lapse movie, related to Figure 5. Excerpt from a 24-h timelapse movie of a *Tg(sox10:lifeact-GFP)* animal with z-stacks collected every 5 min at 59.1 μW starting at 48 hpf.

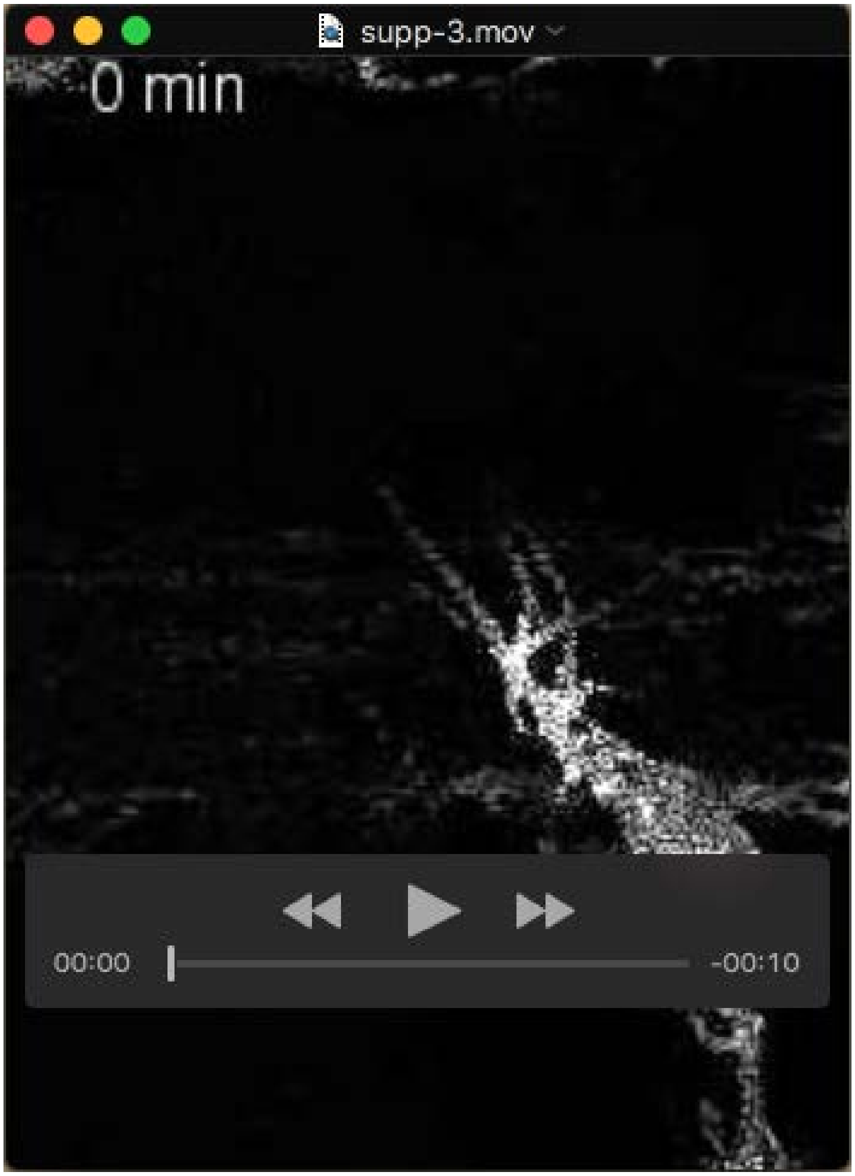

Movie 2. DeSOS time-lapse movie, related to Figure 5. Excerpt from a 24-h timelpase movie of a *Tg(sox10:lifeact-GFP)* animal starting at 48 hpf with z-stacks collected every 5 min and processed for DeSOS.

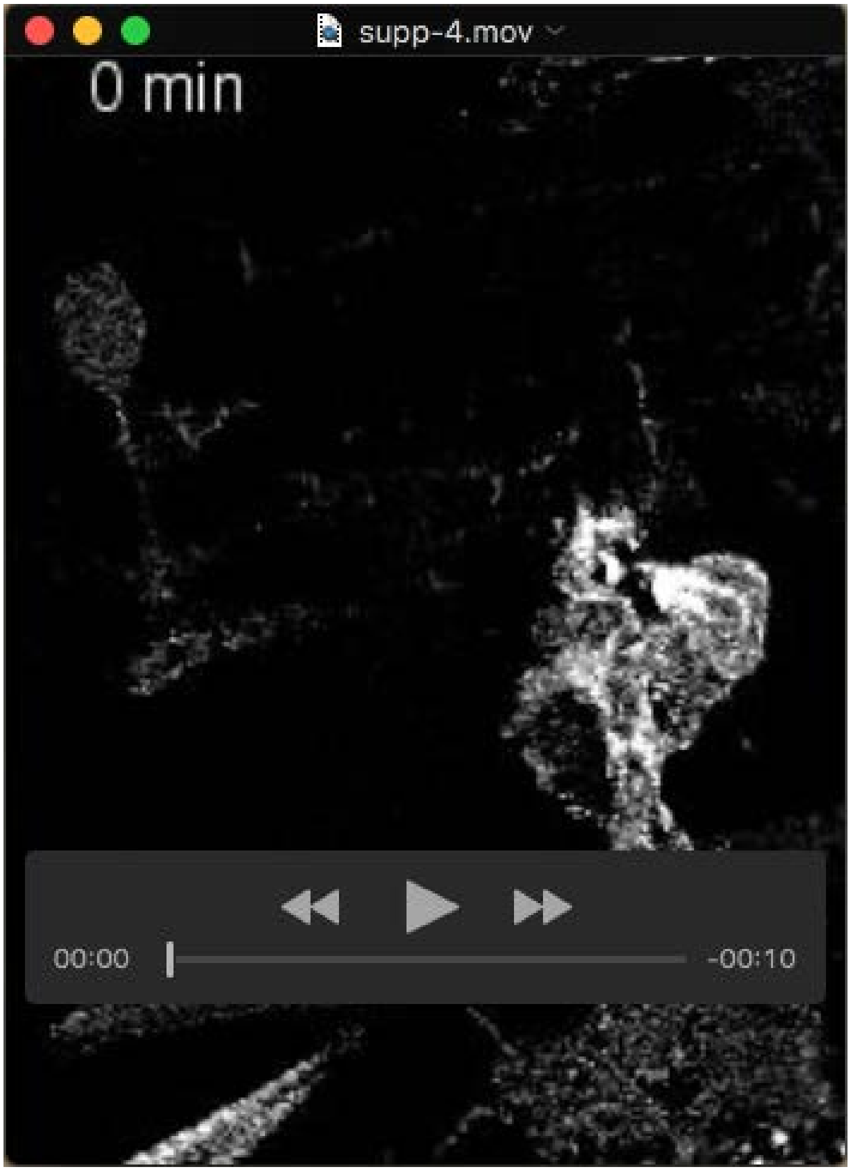

Movie 3. Non-photoactivated DRG display typical pioneer axon selection phenotypes, related to Figure 9. Excerpt from a 24-h DeSOS timelapse movie of a *Tg(sox10:lifeact-GFP); Tg(sox10:PA-Rac1)* animal without exposure to 445 nm light starting at 48 hpf with z-stacks collected every 5 min.

Development • Supplementary information

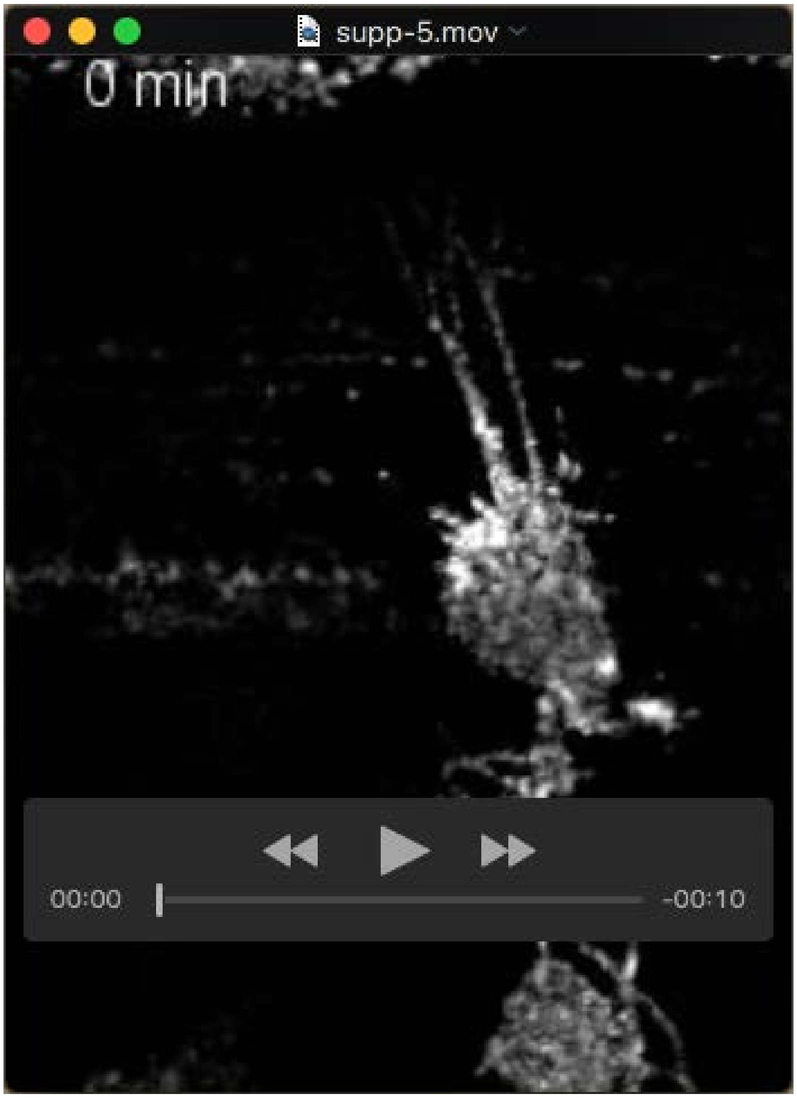

Movie 4. Ectopic Rac1 activation disrupts pioneer axon selection, related to Figure 9. Excerpt from a 24-hr DeSOS timelapse movie of a *Tg(sox10:lifecat-GFP); Tg(sox10:PA-Rac1)* animal exposed to 445 nm light every five minutes. Z-stacks were collected every 5 min starting at 48 hpf. Photoactivation of Rac1 results in a hindered ability for the DRG neuron to select a pioneer axon.

Figure number	Raw Number	n value				p-value	Statistical test
		Number of Animals Represented in Panel	Number of DRG or ROI Represented in Panel	Total Number of Animals Assayed	Number of DRG or ROI Assayed		
1A							
1B							
1C							
1D							
1E							
2A							
2B							
3A		1	1	3	5		
3B		1	1	3	5		
3C		1	1	3	5		
3D		1	1	3	5		
3E	Power 1X: 113.4<br>Power 1.2X: 121.2<br>SOS: 213.8<br>Deconvolution: 100.2<br>DeSOS: 243.2	3	5	3	5	0.0011	One-way paired ANOVA
3F		1	1	2	5		
4A		1	1	3	5		
4B		1	1	3	5		
4C		1	1	3	5		
4D	Power 1X: 46.6<br>Power 1.2X: 58.2<br>SOS: 71.4<br>Deconvolution: 90<br>DeSOS: 116	3	5	3	5	0.0051	One-way paired ANOVA
4E		1	1	2	5		
4F		1	1	6	6		
5A		1	1	3	12		
5B		1	1	3	12		
5C		1	1	3	12		
5D		1	1	3	12		
5E		1	1	3	12		
5F		1	1	3	12		
6A		Photoactivated: 1	Photoactivated: 1	Photoactivated: 5	Photoactivated: 9		
6B		1	1	3	12		
6C	Filopodia: 5.061<br>Basally Projecting Clusters: 0.956	1	1	3	12	<0.0001	Student's t-<br>test
6D	Filopodia: 0.567<br>Basally Projecting Clusters: 1.036	1	1	3	12	<0.0001	Student's t-<br>test
6E		1	1	3	12		
6F		1	1	3	12		
7A		1	1	3	12		
7B		1	1	3	12		
7C		1	1	3	12		
7D		1	1	3	12		
8A							
8B		1	1	3	12		
8C		1	1	3	12		
8D		1	1	6	6		
8E		1	1	6	6		
9A		Non-photoactivated: 1<br>Photoactivated: 1	Non-photoactivated: 1<br>Photoactivated: 1	Non-photoactivated: 3<br>Photoactivated: 3	Non-photoactivated: 8<br>Photoactivated: 11		
9B		1	1	3	8		
9C		1	1	3	11		
9D	Non-photoactivated: 100<br>Photoactivated: 63	Non-photoactivated: 3<br>Photoactivated: 3	Non-photoactivated: 8<br>Photoactivated: 11	Non-photoactivated: 3<br>Photoactivated: 3	Non-photoactivated: 8<br>Photoactivated: 11	<0.0001	Fisher's exact
9E	Non-photoactivated: 18.125<br>Photoactivated: 17.414	Non-photoactivated: 3<br>Photoactivated: 3	Non-photoactivated: 8<br>Photoactivated: 11	Non-photoactivated: 3<br>Photoactivated: 3	Non-photoactivated: 8<br>Photoactivated: 11	0.6935	Student's t<br>test
9F	Non-photoactivated: 27.778<br>Photoactivated: 40.032	Non-photoactivated: 3<br>Photoactivated: 3	Non-photoactivated: 8<br>Photoactivated: 11	Non-photoactivated: 3<br>Photoactivated: 3	Non-photoactivated: 8<br>Photoactivated: 11	0.0157	Student's t<br>test
9G	Non-photoactivated: 297.8<br>Photoactivated: 166.9	Non-photoactivated: 3<br>Photoactivated: 3	Non-photoactivated: 8<br>Photoactivated: 11	Non-photoactivated: 3<br>Photoactivated: 3	Non-photoactivated: 8<br>Photoactivated: 11	0.0396	Student's t<br>test
Supplemental Figures							
S1							
S2A		1	1	3	6		
S2B		1	1	3	5		
S2C		1	1	3	8		
S2D		1	1	3	4		
S3A		1	1	3	5		
S3B		1	1	3	5		
S3C		1	1	3	5		
S3D		1	1	3	5		
S4		1	1	3	12		
S5A		1	1	3	5		
S5B	16	3	5	3	5		
S5C		1	1	3	5		
S5D	28.889	3	5	3	5		
S6A	Non-photoactivated: 10.761<br>Photoactivated: 11.925	Non-photoactivated: 3<br>Photoactivated: 3	Non-photoactivated: 8<br>Photoactivated: 11	Non-photoactivated: 3<br>Photoactivated: 3	Non-photoactivated: 8<br>Photoactivated: 11	0.0076	Student's t<br>test
S6B		1	1	3	8		
S6C		1	1	3	11		
S6D		1	1	3	8		
S6E		1	1	3	11		

Table S1. Summary of statistical measures. Summary of mean values, statistical significance and tests, and number of assayed animals and DRG in each figure.

SUPPLEMENTARY MATERIALS AND METHODS

Standalone DeSOS Microscopy Program.

Executable files can be downloaded to install an application capable of generating DeSOS images. The program and example images can be downloaded from the following links:

https://dx.doi.org/doi:10.7274/r0-5hhg-5578
or
https://curate.nd.edu/show/bz60cv46j4x

Standalone DeSOS Microscopy Program Tutorial.

Guide to generating DeSOS images using the DeSOS Microscopy Program.

1. Take two confocal or two-photon images of your sample. These images should be taken at two different excitation powers. The excitation powers you choose should not obviously saturate your sample.
   a. Be sure to record the excitation powers, in watt, used to take your images. A power meter can be used to do this. The powers should be measured at the focal plane or the back aperture of the objective.
      i. Note that many commercial microscopes use percentages, e.g., 10%, 75%, to quantify the excitation powers in their controlling program settings. In this case, power measurement is still needed because the relationship between the percentages and the actual powers may not be linear, i.e., the ratio between the percentages and their corresponding excitation powers is not a constant. However, if the measurements show that the relationship is linear and the ratio is a constant, then the percentages can be directly used as the power parameters in the DeSOS program.
   b. In addition, you should record the following image parameters of your system. These are required for the DeSOS program to process your images.
      i. wavelength of the fluorescence, in nanometers.

Development • Supplementary information

ii. numerical aperture of the objective lens.

iii. refractive index of the immersion oil or air.

iv. image slice pixel width (x- and y-direction), in nanometers.

v. image stack voxel depth (z-direction), in nanometers.

2. Install the DeSOS program on a Windows PC.

   a. If your PC has Matlab installed, extract all the content from "DeSOS_Matlab_Application.zip". Run the file "StartDeSOS.m" with Matlab to start the program. (*We recommend this approach because it greatly saves your disk space, with a size of <200 kB.*)

   b. If your PC does not have Matlab installed, open the executable file "DeSOS_Standalone_Application.exe". Follow instructions to finish the installation. Once installed, use the shortcut "DeSOS_Microscopy" on your desktop to start the program. *(This approach could take ~1GB of your disk space because it requires additional download and installation of Matlab runtime environment.)*

3. You will see a window with empty axes as shown below.

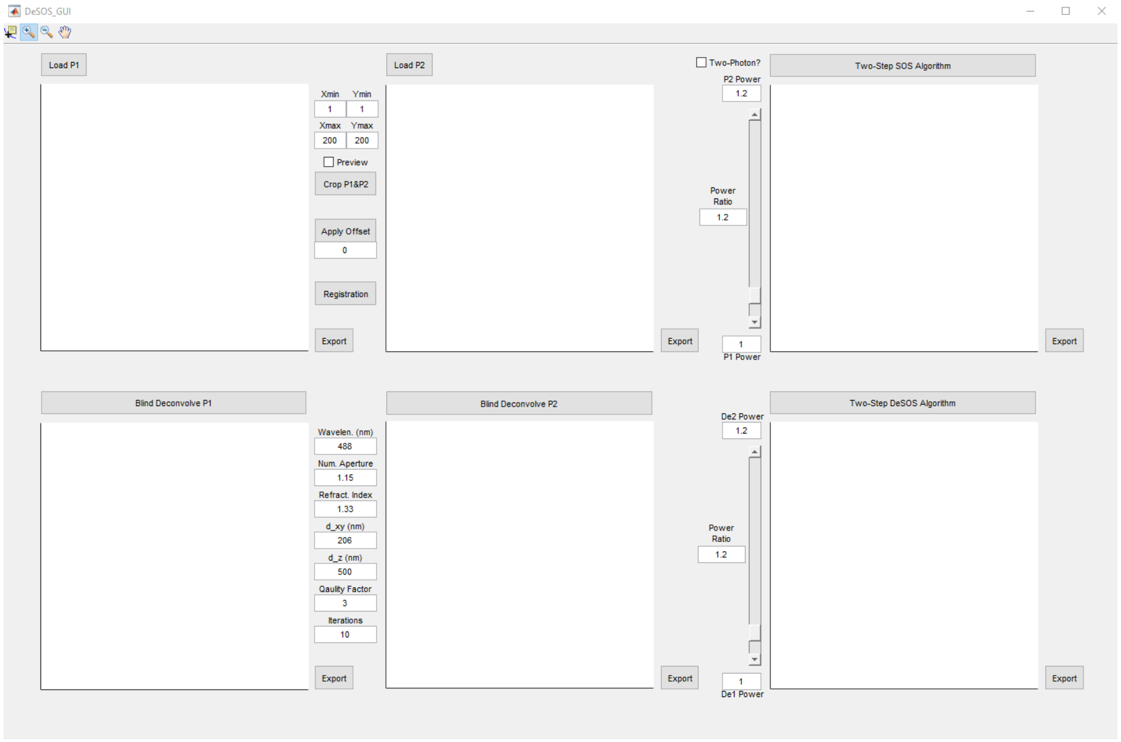

4. Click “Load P1” and “Load P2” to load the first- and second-step images that you want to process with the SOS/DeSOS algorithm. Generally, “P1” is obtained with a lower excitation laser power compared to the power for “P2”. For example, in the screenshot below, two example images “Power=75Percent_3D.tif” and “Power=76Percent_3D.tif” are loaded into the program.

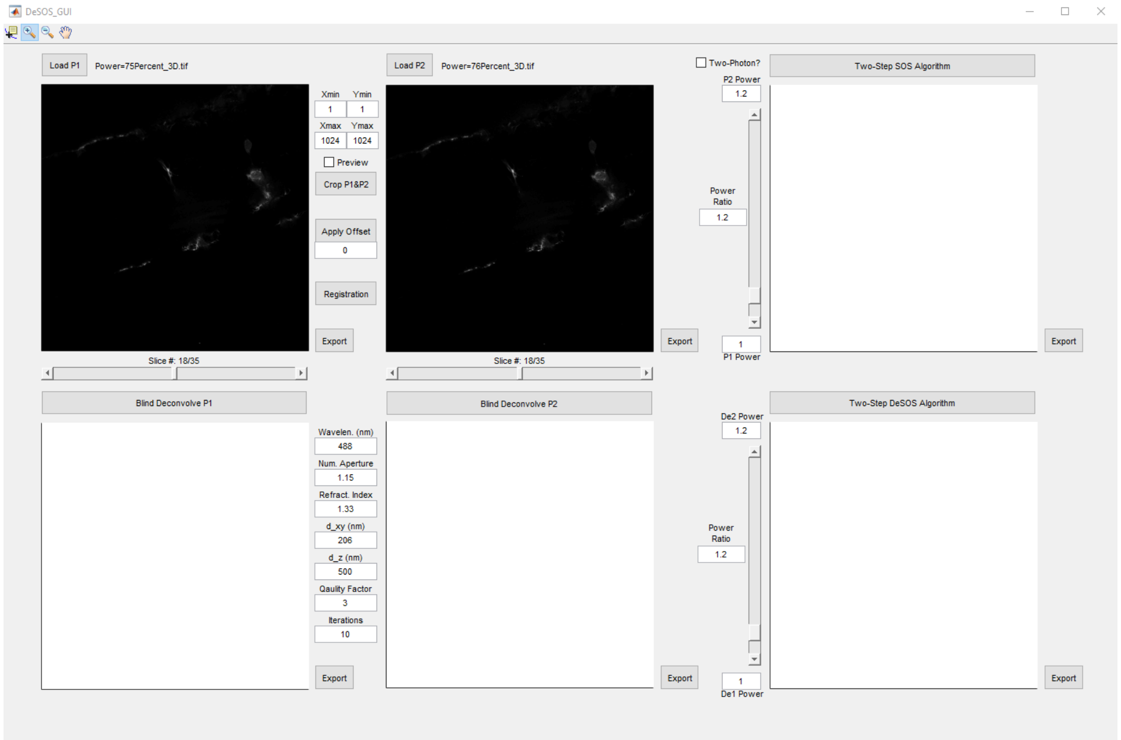

a. The program allows for the import of both 2D and 3D images.

b. For DeSOS algorithm, 3D image stacks are recommended because the blind deconvolution works better with 3D image stacks. Though 2D blind deconvolution is also viable, 3D deconvolution allows for the removal of out-of-focus blur, hence providing better performance.

c. If the images are 3D stacks, the user can use the sliders below the images to change the slice that is currently being displayed. The current and total slice number will be shown above the slider.

d. *(Optional)* “P1” and “P2” images can be cropped (on XY plane) simultaneously according to user-defined cropping boundaries, “Xmin”, “Xmax”, “Ymin”, “Ymax”. Specifically, [Xmin, Ymin] and [Xmax, Ymax] are the coordinates of the top-left and bottom-right corners of the cropping region on the original image plane (before

Development • Supplementary information

cropping). Check the box “Preview” to preview the cropping operation. If not satisfied, uncheck “Preview”, change the cropping boundaries, and repeat this process; if satisfied, click “Crop P1&P2” to perform the cropping operation. A message box will appear to confirm the operation, click “Yes” to proceed; this operation cannot be undone, so if the user wants to reverse or change the cropping operation, they will need to reload the raw images.

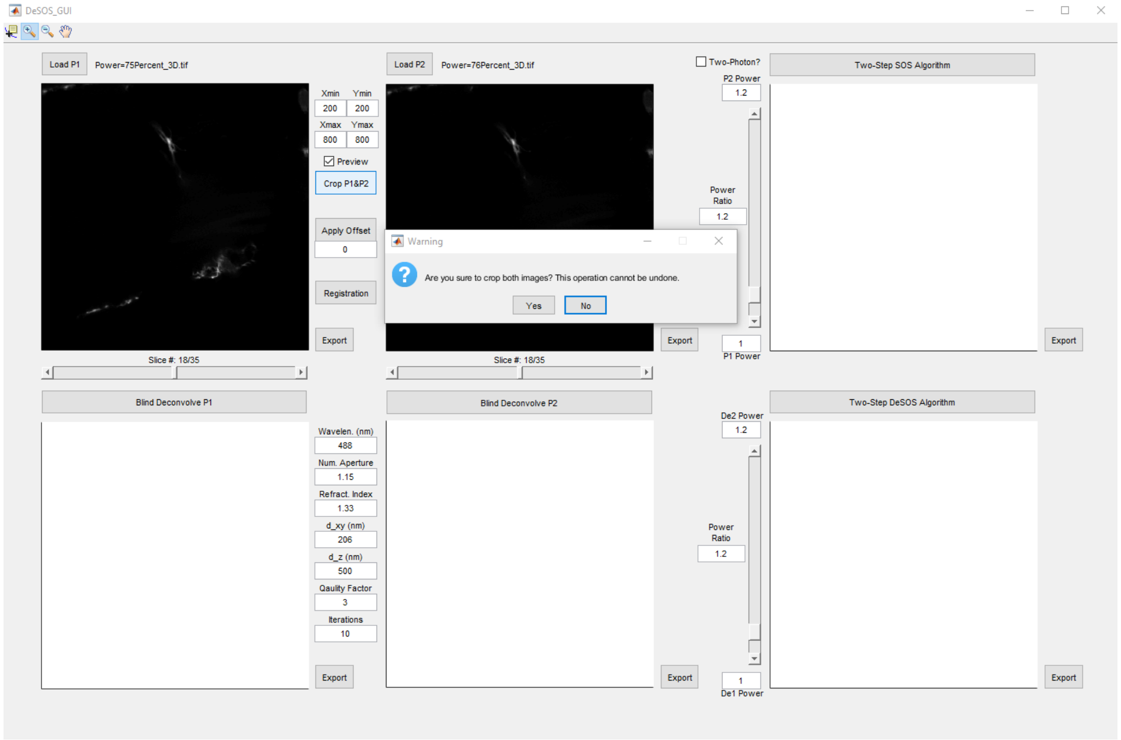

e. *(Optional)* An offset can be applied to all pixel values on both images. This can be used when your images have a constant background value, e.g., 50, on all pixels; i.e., even the pixels appear to be totally black or empty, they still process a value of 50. This is mainly caused by the offset settings on the detector when acquiring these images. Note that this value is different from background noise. In SOS/DeSOS, a constant background offset (like the constant pixel value of 50 here) could introduce artifacts. It could be eliminated by clicking the button “Apply Offset” with a value “-50” filled in the box below.

f. *(Optional)* SOS/DeSOS requires that images “P1” and “P2” are taken from the same field of view. In practice, it may be hard to meet this requirement considering that sample drift could happen when acquiring images. To eliminate this problem, the program provides an image registration tool to register “P2” using “P1” as a reference; i.e., it calculates the drift between “P2” and “P1” and then moves “P2” accordingly to cancel this drift. When image registration is complete, there should be no obvious drift between the two images. To perform this operation, click the button “Registration”. A message will appear to confirm this operation. Click “Yes” to proceed. Depending on the size of the images, this operation could take a while. A message box showing the registration progress will show up.

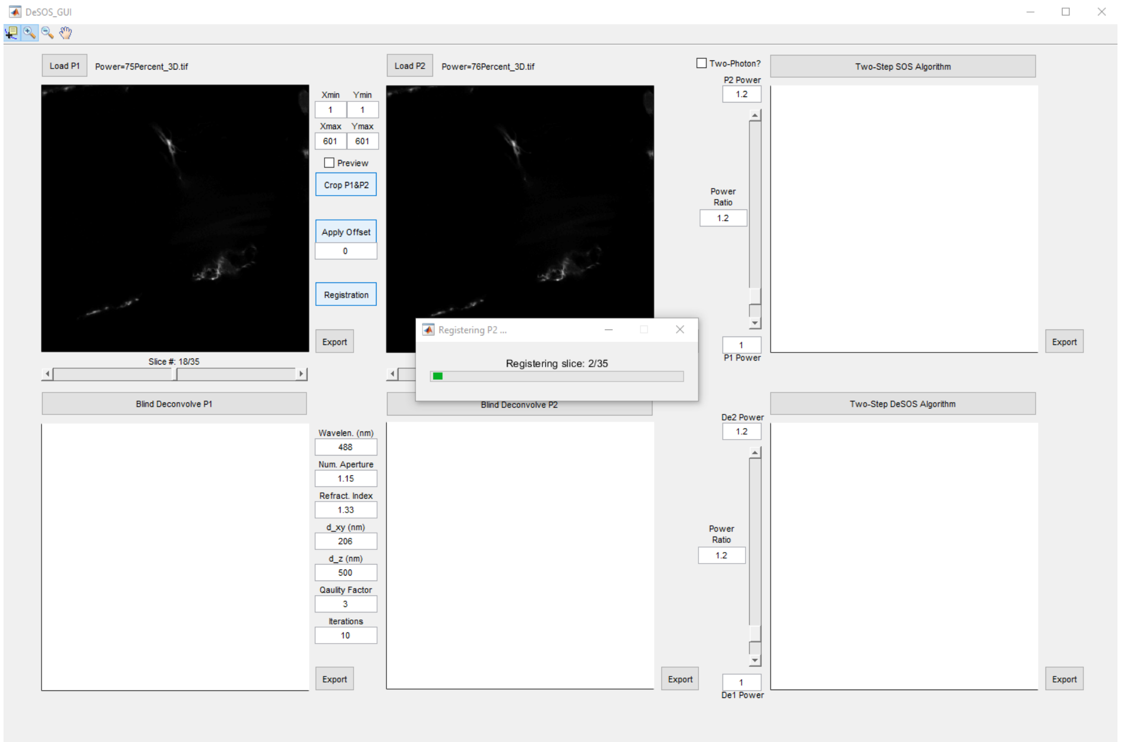

5. Input “P1 Power” and “P2 Power” with the excitation powers (measured with a power meter) that you used to obtain images “P1” and “P2”, respectively. Alternatively, you can just input either one of “P1 Power” and “P2 Power” and fill in “Power Ratio”; the other power parameter will be calculated automatically. For example, in the screenshot below, we input “76” for “P2

Development • Supplementary information

Power" and "75" for "P1 Power" because "Power=76Percent_3D.tif" was obtained with a power setting of "76%" and "Power=75Percent_3D.tif" with "75%" (the power percentages of our microscope are linear related to the measured excitation powers).

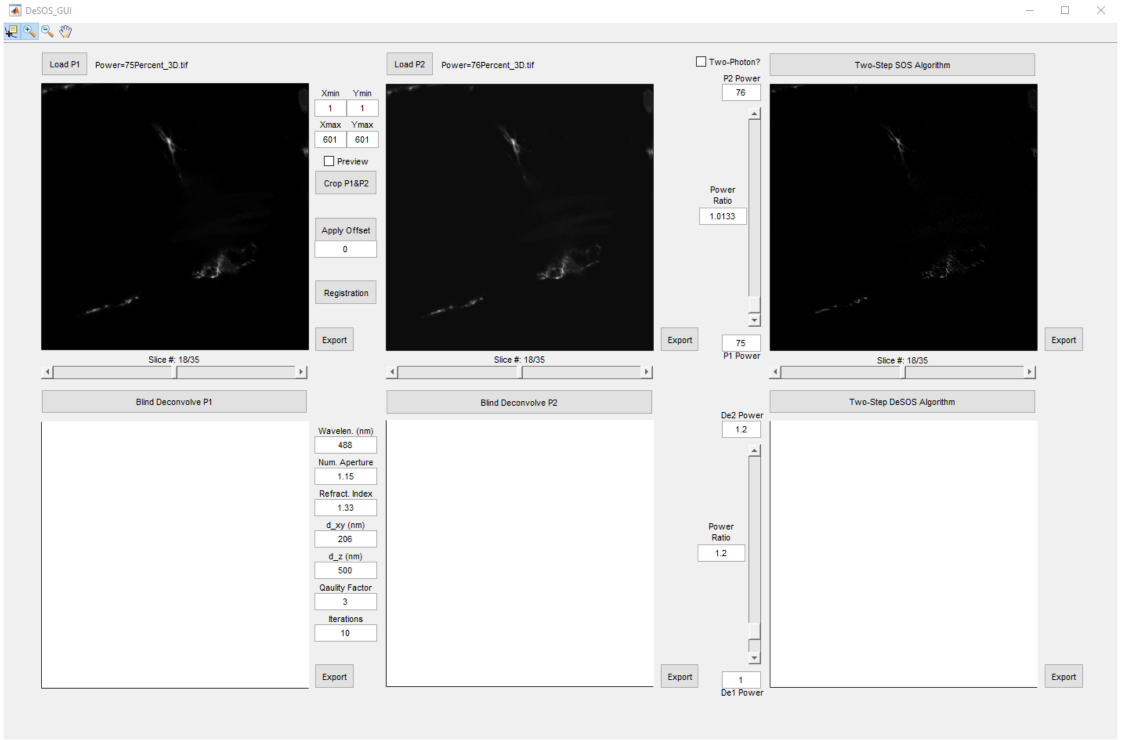

6. Check "Two-Photon" box if the images are obtained using a two-photon microscope. If the images are obtained using a confocal microscope, uncheck the box. The example images were acquired with a spinning disk confocal microscope; therefore the "Two-Photon" box is unchecked.
7. Click "Two-Step SOS Algorithm" to perform SOS algorithm on images "P1" and "P2". The resulting image "SOS" will show up.
8. Input the blind deconvolution parameters of the raw images "P1" and "P2". Theoretically, no parameters would be necessary for blind deconvolution, because the algorithm itself can estimate the optimal point spread functions (PSFs) and deconvolved images. In practice, however, the iterative blind deconvolution algorithm that we use might require an extensive amount of computational time to find an optimal solution, and sometimes this solution is simply

Development • Supplementary information

a “local minimum”. Therefore, an initial estimation of the PSFs as accurate as possible is recommended. In this program, we calculate the theoretical PSFs based on diffraction theory using the imaging parameters from the user. The calculated PSFs are used as an initial estimation for the iterative blind deconvolution algorithm. These parameters are:

a. “Wavelen. (nm)”: wavelength of the fluorescence, in nanometers.
b. “Num. Aperture”: numerical aperture of the objective lens.
c. “Refract. Index”: refractive index of the immersion media or air.
d. “d_xy (nm)”: image slice pixel width (x- and y-direction), in nanometers.
e. “d_z (nm)”: image stack voxel depth (z-direction), in nanometers.
f. “Quality Factor”: an empirical factor (≥1, usually 1 to 3) used to account for nonidealities such as spherical aberrations and refractive index mismatch when calculating the initial PSFs. This parameter could be varied to adjust the deconvolution performance. If the deconvolution performance is not satisfactory, for example, the contrast or signal-to-noise ratio is getting even worse than the raw images, the user can change this quality factor to variate the performance.
g. “Iterations”: the number of iterations of the iterative blind deconvolution algorithm. More iterations could have better performance but will take longer computational time. A value of “20” is recommended for most cases.

9. Click “Blind Deconvolve P1” to perform iterative blind deconvolution algorithm on image “P1”. When it is done, click “Blind Deconvolve P2” to deconvolve image “P2”. This step might be time-consuming. For a 2D image, it could take a few seconds; for a 3D stack, it could take minutes for an iteration number “N_int” of 20. A message box will appear to show the deconvolution progress.

Development • Supplementary information

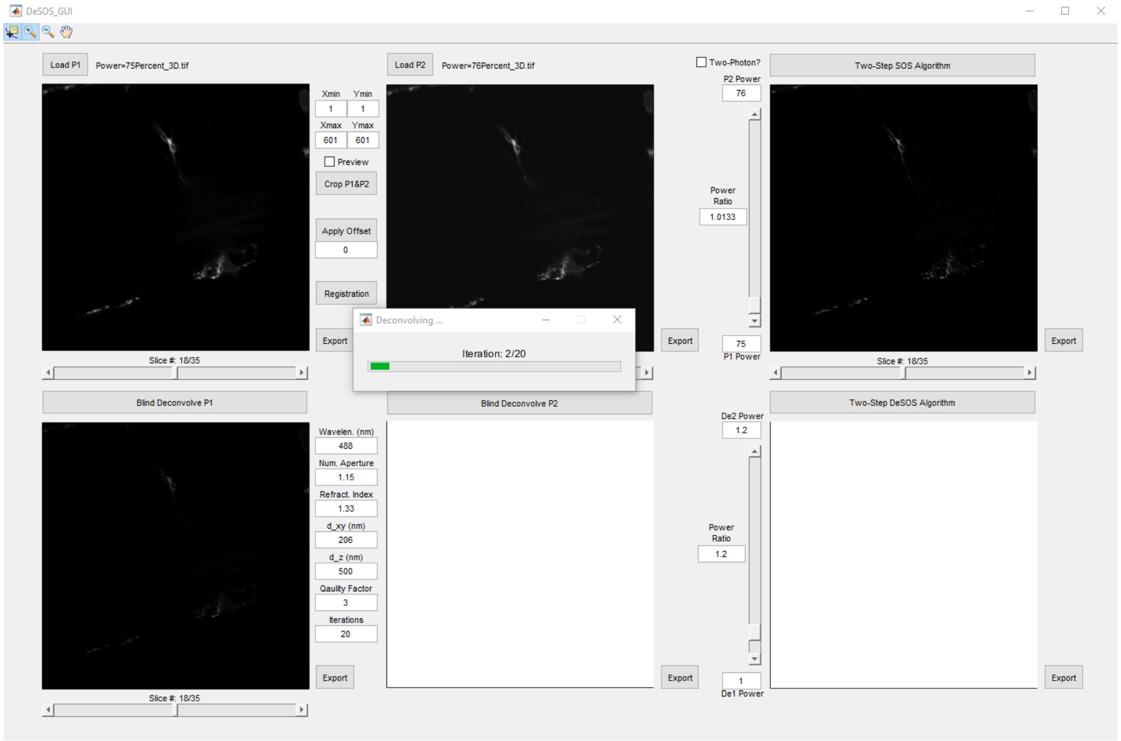

10. Once the blind deconvolution is done for both images, two deconvolved images “De1” and “De2” corresponding to “P1” and “P2”, respectively, will show up.
11. Identical to procedure #4, input “De1 Power” and “De2 Power” with the excitation powers corresponding to “De1” and “De2”. Alternatively, you can just input either one of “De1 Power” and “De2 Power” and fill in “Power Ratio”; the other power parameter will be calculated automatically. For this example, we input “76” for “De2 Power” and “75” for “De1 Power”, identical to the power setting for SOS algorithm.
12. Identical to procedure #5, check “Two-Photon” box if the images are obtained using a two-photon microscope; uncheck the box if the images are obtained with a confocal microscope.
13. Click “Two-Step DeSOS Algorithm” to perform DeSOS algorithm on deconvolved images “De1” and “De2”. The result image “DeSOS” will show up.

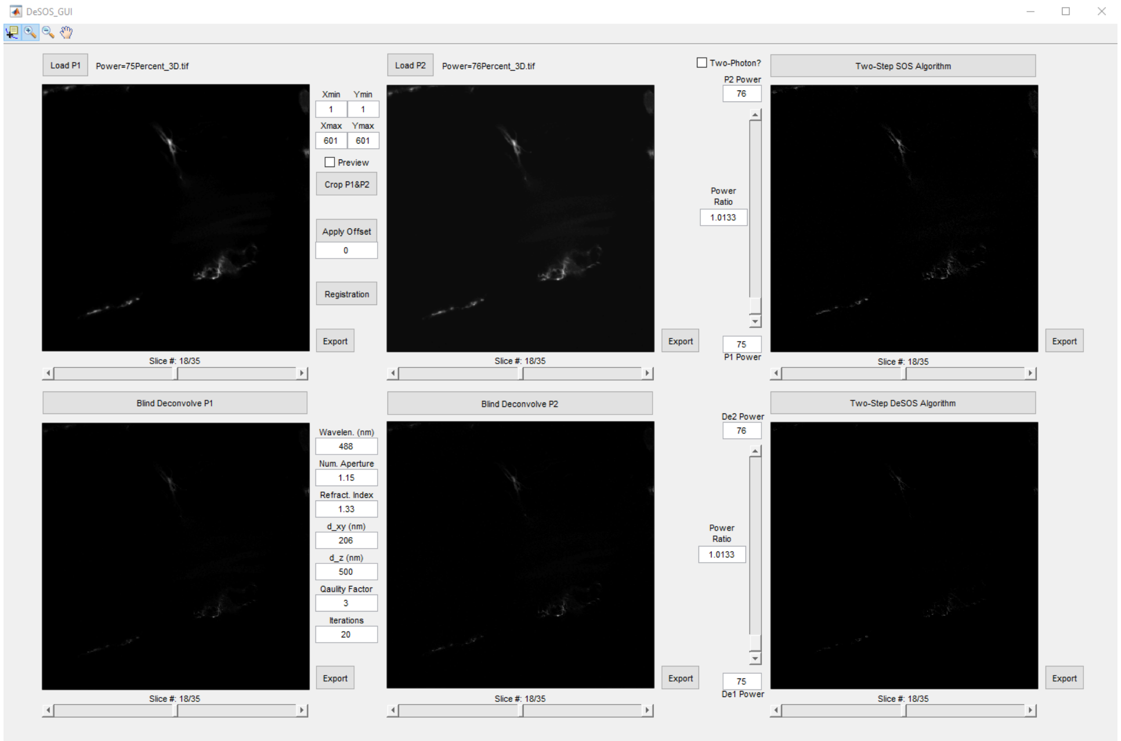

14. The “Export” buttons next to each image can be used to export the corresponding images to 32-bit (single precision) TIF files. The program will save 2D and 3D images as TIF images and stacks respectively.
15. The icons on the toolbar can be used to add cursor, zoom in, zoom out, and pan any images in the program. For example, we can zoom in all images to show the resolution improvement of the SOS/DeSOS algorithm.
    a. Note that the brightness of SOS/DeSOS images may not be high enough to show its structural details. We suggest users to export the images first, and then view them and adjust their brightness/contrast in another program, e.g., ImageJ, to observe the details. For 3D stacks, a max z-projection is a good way to visualize the resolution improvement. The screenshot below shows the max z-projections of the exported raw image stack “P1” (left) and the resulting DeSOS stack (right). The brightness/contrast has been adjusted to visualize the details. Compared to the raw image, the improved resolution of the DeSOS image can be clearly seen.

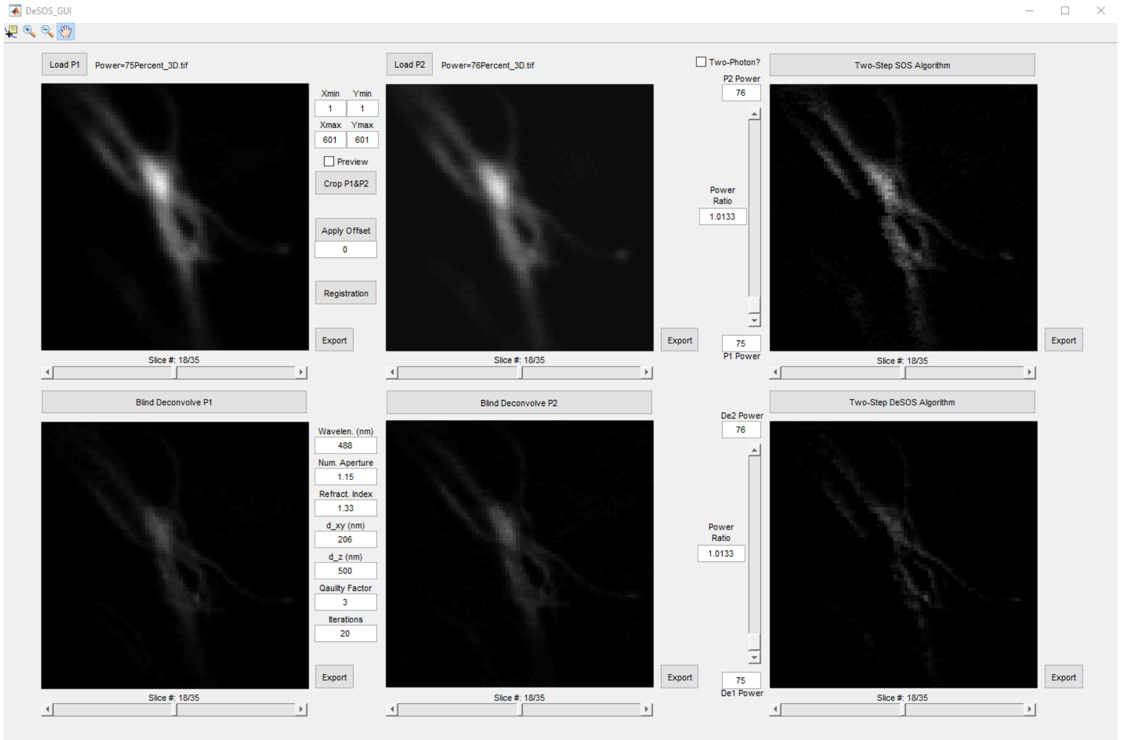

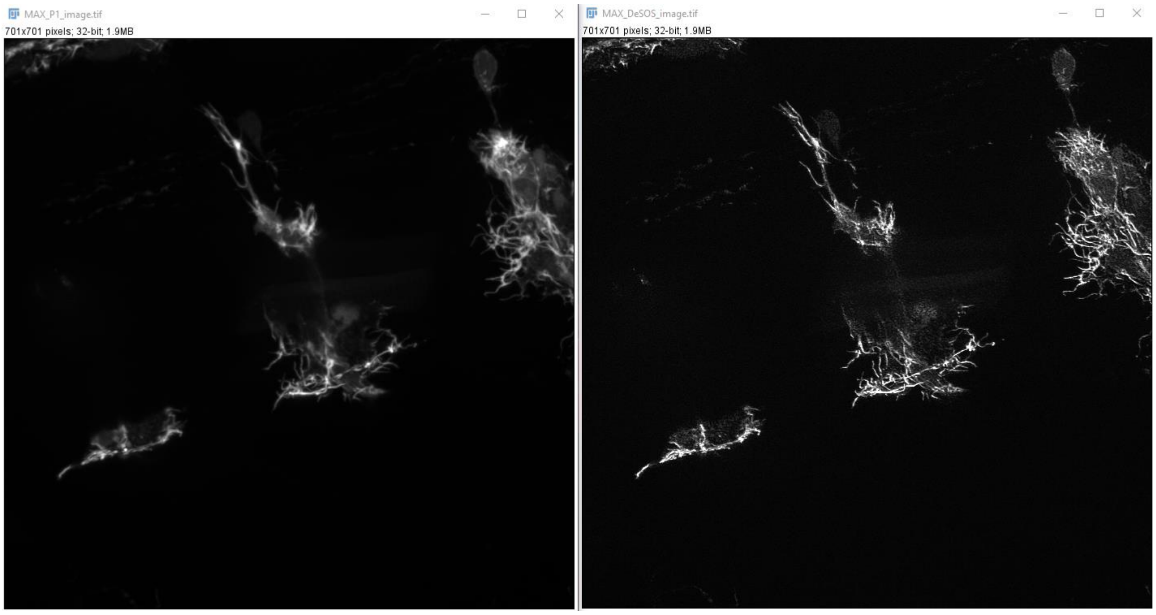

16. The user is encouraged to try the two sets of example images in the installation package.

a. The first set is a 2D image pair, “Power=2.8uW_2D.tif” and “Power=3.2uW_2D.tif”. They are scanning confocal 2D fluorescent images of Alexa Fluor 488 phalloidin labeled F-actin in fixed endothelial colony forming cells (ECFCs). They are obtained with a 100x, 1.45 NA, oil-immersion (refractive index of 1.515) objective and the pixel

width (d_xy) is 59 nm. “Power=2.8uW_2D.tif” is obtained with an excitation power of 2.8 μW, and “Power=3.2uW_2D.tif” is obtained with 3.2 μW.

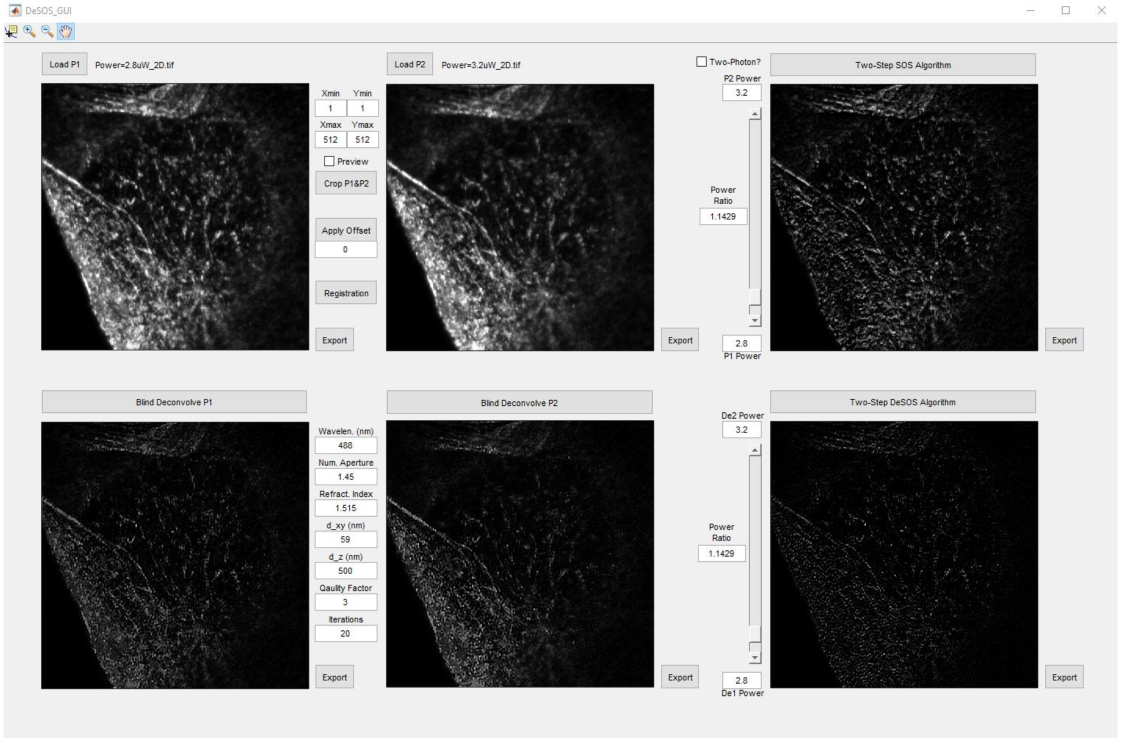

b. The second set is a 3D stack pair, “Power=75Percent_3D.tif” and “Power=76Percent_3D.tif”. They are spinning disk confocal 3D fluorescent stacks of EGFP in genetic modified zebrafishes. They are obtained with a 63x, 1.15 NA, water-immersion objective. The pixel width (d_xy) is 206 nm, and the voxel depth (d_z) is 500 nm. “Power=75Percent_3D.tif” is obtained with a laser power setting of 75%, and “Power=76Percent_3D.tif” is obtained with 76%. They have been used as the example throughout this tutorial.

c. Note that the SOS/DeSOS algorithm relies on the relative ratio of excitation powers for the two images; therefore, it is not always necessary to measure the exact powers. It is valid for us to use power percentages (75%, 76%), instead of actual measured powers, to parameterize our algorithm because we have already experimentally

verified that our power percentages are linear (no bias) related to the actual powers. Nevertheless, measuring actual powers as parameters is always the safest choice.

d. Also note that photobleaching of fluorophores could be inevitable since two images should be obtained on the same region of interest. In this case, the fluorescence intensity of the second image could be lower than what it is supposed to according to its excitation laser power due to the loss of fluorophores. If this is indeed an issue, the user is recommended to manually adjust the power ratio using the slider next to it until a satisfactory SOS/DeSOS image is generated.

Experimental Model and Subject Details

Zebrafish strains were as follows: *Tg(sox10:gal4)*(Hines et al., 2015), *Tg(uas:lifeact-gfp)*(Helker et al., 2013), *Tg(sox10:mrfp)*(Kucenas et al., 2008), *Tg(phox2b:gal4)*(Hines et al., 2015), *Tg(uas:syn-gfp)*(Heap et al., 2013). Pairwise matings were used to produce embryos. Embryos of either sex were used for all experiments. Stable, germline transgenic lines were used for all analysis.

Cx3cr1GFP/+ mice were generated in house by breeding B6.129P-Cx3cr1tm1Litt/J (005582/CX3CR1-GFP) mice to C57BL/6 (000664/Black 6) mice, which were both purchased from Jackson Lab (Ben Harbor, ME). Females were used for this study.

In vivo imaging with spinning disk confocal microscopy of zebrafish

Embryos were manually dechorionated at 48 hpf and anesthetized with 3-amino-benzoic acid ester (Tricaine). Anesthetized embryos were immersed in 0.8% low-melting point agarose and mounted on their right side in glass-bottomed 35 mm Petri dishes. A spinning disk confocal microscope from 3i technology© was used for all imaging. A Zeiss Axio Observer Z1 Advanced Mariana Microscope was equipped with X-cite 120LED White Light LED System and filter cubes for GFP and mRFP, a motorized X,Y stage, and a piezo Z stage. The microscope has three objectives: a 20X Air (0.50 NA) objective with a working distance of 2 mm, 63X (1.15NA) water objective with a working distance of 0.66 mm, and 40X (1.1NA) water objective with a working distance of 0.62 mm. The microscope also has a CSU-W1 T2 Spinning Disk Confocal Head (50 uM) with a 1X

Development • Supplementary information

camera adapter and andor iXon3 1Kx1K EMCCD camera as well as dichroic mirrors for 446, 515, 561, 405, 488, 561, 640 excitation and a laser stack containing 405 nm, 445 nm, 488 nm, 561 nm and 637 nm with laserstack FiberSwitcher with 250 uS switch time, photomanipulation with vector© high speed diffraction-limited point scanner ablations, and Ablate!TM© Photoablation System (532 nm pulsed laser with a pulse energy 60J @ 200 HZ). Time lapse images were taken every 5 minutes for 24 hours starting at 48 hpf. Adobe Illustrator and ImageJ were used to process images and enhance image brightness and contrast.

Intravital Microscopy of Mice

Intravital imaging of the mouse brain with two-photon microscopy was performed similarly to previously described (Grutzendler et al., 2002). Briefly, Cx3cr1-GFP/+ mice were anesthetized with ketamine and xylazine cocktail by intraperitoneal injection. The mouse's head was secured and fixed in place using a stereotaxic instrument (Stoelting Co), and the skull was exposed with a midline scalp incision. A high-speed microdrill (Ideal Microdrill) equipped with a 0.7mm burr (Fine Science Tools, 19007-09) was used to thin the skull to approximately 30µm in thickness, using light sweeping motions to thin the skull gradually without applying significant pressure to the brain. PBS was applied to the thinned skull to enhance transparency for imaging.

Following surgery, the anaesthetized mouse was immediately imaged on an Olympus FV1000 microscope equipped with tungsten and halogen visible light sources, four lasers (argon 458nm, 488nm, 515nm; HeNe 543 nm; red diode 635; and a Mai Tai DeepSee

Development • Supplementary information

titanium-sapphire 690-1040nm), and a cube filter set with the following spectral configurations: 460-500, 520-560, 575-625, 650-700. All imaging employed a 25× objective water immersion objective (XLSLPLN25XGMP, Olympus USA; NA = 1.0 and WD = 8 mm). The laser was tuned to the excitation wavelength for GFP (920 nm) at four step-wise tuned laser powers (9.37 mW, 10.68 mW, 11.95 mW, and 13.25 mW), and a digital zoom was set to (4x). The imaging depth was ~100µm from the pial surface and when Z-stacks were acquired, the step size was 1 µm.

DeSOS microscopy

The blind deconvolution operation can be performed using various commercial or open-access programs. In this work, the images were blind deconvolved using either AutoQuant Blind or a blind deconvolution algorithm implemented in Matlab. Both blind deconvolution methods utilized iterative maximum likelihood estimation (MLE) algorithms, which can be explained as follows (Biggs and Andrews, 1997; Holmes et al., 2006). The imaging process is modeled as $g(r) = h(r) \otimes f(r) + n(r)$, where r denotes the 3D spatial coordinate, $g(r)$ the measured distorted image, $f(r)$ the ideal undistorted image, $h(r)$ the PSF of the system, $\otimes$ the inherent convolution process, and $n(r)$ the noise. MLE iteratively estimates $h(r)$ and $f(r)$ simultaneously which have the highest likelihood of being correct given the measured imaging data. Specifically, the algorithm first guesses an initial $h(r)$, and then estimate which $f(r)$ could have generated $g(r)$. The estimated $f(r)$ is then reblurred by $h(r)$ and compared to the actual image $g(r)$, where the error in this comparison is used to re-estimate $h(r)$ in order to reduce the error in estimation. These steps are repeated again and again until a convergence is reached or a certain

stopping criterion is satisfied. For the deconvolution with AutoQuant Blind, the images were imported, deconvolved, and exported using the commercial software; for the deconvolution with Matlab, the MLE algorithm was integrated in our open-access DeSOS application which could perform both blind deconvolution and SOS operations.

The second operation of DeSOS microscopy, SOS, is a saturation-based super-resolution fluorescence microscopy technique that can be easily implemented and requires no additional hardware. In general, SOS linear combines M conventional (confocal or two-photon) fluorescence images to generate a super-resolved image with a $\sqrt{M}$-fold resolution improvement compared to the diffraction limit. The method is based on the steady-state solution of fluorescence intensity in a two-level fluorophore model, $H(r) = K[aI^N G^N(r)]/[1 + aI^N G^N(r)]$, where r is the spatial coordinate, I the peak excitation intensity, $G(r) = \exp(-2r^2/\omega_0^2)$ a Gaussian excitation profile with a $1/e^2$ radius ω_0^2, N the number of excitation photons needed for a fluorophore to emit one photon ($N = 1$ for confocal, $N = 2$ for two-photon), $H(r)$ the effective PSF of the system, and a and K the constants related to detection efficiency, cross-section, excitation wavelength, etc. Note that the excitation profile $G(r)$ is assumed to be Gaussian to simplify the illustration; in practice, $G(r)$ can be of any other mathematical forms that can describe the focus intensity distribution, e.g., Lorentzian, and SOS will still work. SOS utilizes the Taylor expansion of $H(r)$, which is $H(r) = K[aI^N G^N(r) - a^2 I^{2N} G^{2N}(r) + a^3 I^{3N} G^{3N}(r) - \cdots]$. Considering the (Gaussian) excitation profile $G(r)$, high powers of $G^N(r)$, such as $G^{2N}(r)$, $G^{3N}(r)$, etc., represent components with higher spatial frequency (resolution), while $G^N(r)$ itself is diffraction-limited; an M-th order power component, $G^{MN}(r)$, has a $\sqrt{M}$-fold

Development • Supplementary information

increase in spatial resolution. However, due to magnitude difference among each components, the spatial resolution of $H(r)$ is dominated by the lowest power of $G^N(r)$; hence it is diffraction-limited. The concept of SOS is to eliminate the lowest $M-1$ powers of $G^N(r)$ by linear combining M conventional images (confocal or two-photon), $F_1, F_2, \cdots, F_M$, obtained at difference excitation intensities, $I_1, I_2, \cdots, I_M$; the resulting SOS image therefore will have an effective PSF generated from the linear combination of $H_1(r), H_2(r), \cdots, H_M(r)$, i.e., $H_{MSOS}(r) = \sum_{i=1}^{M} c_i H_i(r)$, where the coefficients c_i are chosen such that the lowest power (dominant component) in $H_{MSOS}(r)$ is $G^{MN}(r)$, a $\sqrt{M}$-fold increase in resolution. The coefficients c_i for different types of SOS methods and how they are calculated can be found in (Zhang et al., 2018). For two-step SOS ($M = 2$), the linear combination coefficients are $c_1 = 1$ and $c_2 = -I_1^N/I_2^N$, and the $\sqrt{2}$-fold resolution improvement can be seen from the analytical form of $H_{2SOS}(r)$, i.e., $H_{2SOS}(r) = K[-a^2 I_1^N(I_1^N - I_2^N)G^{2N}(r) + a^3 I_1^N(I_1^{2N} - I_2^{2N})G^{3N}(r) - \cdots]$, which is dominated by the second order component $G^{2N}(r)$. In this work, we performed two-step SOS only because (a) more steps ($M > 2$) required repetitive imaging acquisition on the same field of view, which could cause severe photobleaching problems that hinder the SOS performance, and (b) the coefficients c_i for three-step (or more) SOS could be hard to be accurately determined due to their more complicated forms, e.g., in three-step SOS, $c_1 = 1$, $c_2 = -[I_1^N(I_1^N - I_3^N)]/[I_2^N(I_2^N - I_3^N)]$, $c_3 = [I_1^N(I_1^N - I_2^N)]/[I_3^N(I_2^N - I_3^N)]$, and the errors in measuring I_i. We used a digital optical power meter (Thorlabs PM100D) and a detector (Thorlabs S120C) to measure the laser power at the sample plane for each step of the two-step SOS. For the deconvolution with AutoQuant Blind, the deconvolved images were imported to Matlab and the SOS operation was performed with a script; for the

Development • Supplementary information

deconvolution with Matlab, SOS was integrated in our open-access DeSOS application and the images deconvolved with the MLE algorithm were automatically processed with the SOS algorithm. All output images were exported as TIF files for further analysis.

Simulation

The excitation profile was calculated as $G(u,v) = \left|2\int_0^1 J_0(v\rho)\exp(iu\rho^2/2)\rho d\rho\right|^2$, where J_0 was the Bessel function of the first kind with order zero; u and v were axial and radial normalized optical coordinates, which were related to the real axial and radial coordinates, z and r, by $u = 8\pi zn\sin^2(\alpha/2)/\lambda$ and $v = 2\pi rn\sin\alpha/\lambda$; $n\sin\alpha$ was the numerical aperture of the objective, n the refractive index of the immersion medium, and λ the wavelength. The saturable excitation PSF was then calculated as $H(u,v) = K[aI^N G^N(u,v)]/[1 + aI^N G^N(u,v)]$ and the confocal PSF was obtained as $H(u,v) \times G(u,v)$. For the PSF simulation, two sets of confocal PSFs were simulated with different excitation intensities I_1 and I_2 corresponding to the 1X and 1.2X the laser power. For the two-point objects simulation, the simulated confocal PSFs were convolved with several two-point structures where the distance between the two points were different, e.g., 0.3X or 0.35X the excitation wavelength. The simulated PSFs and images were exported as TIF files and imported to AutoQuant Blind for deconvolution. All simulated and deconvolved PSFs and images were then imported to Matlab for SOS and DeSOS processing. The simulation parameters were as follows: illumination and detection wavelength, 488 nm; numerical aperture, 1.1; refractive index, 1.33; lateral pixel size, 5 nm; axial slice depth, 5 nm; voxel numbers, 256×256×256. The simulated two-point

Development • Supplementary information

objects were visualized with 3D isosurfaces to demonstrate the differentiability of each imaging modality.

Quantification and Statistical Analysis

Pixel groupings were completed using two distinct methods. Composite outline renderings of distinct actin populations were created by first dividing the range of pixel intensities into quartiles. Distinct pixel populations were then visualized by adjusting the intensity threshold of the image to reflect a particular quartile. Particle analysis was then completed to develop the outline rendering of each quartile. These renderings were then compiled into a single composite image.

Grouping of pixels to determine the area and intensity of distinct groupings was completed using ImageJ's magic (tracing) tool. First the tolerance of this tool was set to 10% of the maximum pixel intensity in the image. (E.g. If the maximum pixel intensity in the image was 1000 au, then the threshold would be set at 100 au.) This change in tolerance allows for ImageJ to select adjacent pixels that have intensity values within 100 au of each other. Thorough pixel groupings using the magic (tracing) tool were completed for distinct ROI in the image. The location, size, and area of each grouping were recorded. The location of each group was used to prevent counting pixel groupings more than once.

SUPPLEMENTAL REFERENCES

Biggs, D. S. C. and Andrews, M. (1997). Acceleration of iterative image restoration algorithms. *Appl. Opt.* **36**, 1766.

Grutzendler, J., Kasthuri, N. and Gan, W. B. (2002). Long-term dendritic spine stability in the adult cortex. *Nature* **240**, 812–816.

Heap, L. A., Goh, C. C., Kassahn, K. S. and Scott, E. K. (2013). Cerebellar Output in Zebrafish: An Analysis of Spatial Patterns and Topography in Eurydendroid Cell Projections. *Front. Neural Circuits* **7**, 53.

Helker, C. S. M., Schuermann, A., Karpanen, T., Zeuschner, D., Belting, H.-G., Affolter, M., Schulte-Merker, S. and Herzog, W. (2013). The zebrafish common cardinal veins develop by a novel mechanism: lumen ensheathment. *Development* **140**, 2776–86.

Hines, J. H., Ravanelli, A. M., Schwindt, R., Scott, E. K. and Appel, B. (2015). Neuronal activity biases axon selection for myelination in vivo. *Nat. Neurosci.* **18**, 683–689.

Holmes, T. J., Biggs, D. and Abu-Tarif, A. (2006). Blind Deconvolution. In *Handbook Of Biological Confocal Microscopy*, pp. 468–487. Boston, MA: Springer US.

Kucenas, S., Takada, N., Park, H. C., Woodruff, E., Broadie, K. and Appel, B. (2008). CNS-derived glia ensheath peripheral nerves and mediate motor root development. *Nat. Neurosci.* **11**, 143–151.

Zhang, Y., Nallathamby, P. D., Vigil, G. D., Khan, A. A., Mason, D. E., Boerckel, J. D., Roeder, R. K. and Howard, S. S. (2018). Super-resolution fluorescence microscopy by stepwise optical saturation. *Biomed. Opt. Express* **9**, 1631–1629.

Development • Supplementary information
